# *In Vitro* Pharmacological Profile of a New Small Molecule Bradykinin B_2_ Receptor Antagonist

**DOI:** 10.3389/fphar.2020.00916

**Published:** 2020-06-19

**Authors:** Anne Lesage, Christoph Gibson, François Marceau, Horst-Dieter Ambrosi, Jörn Saupe, Werner Katzer, Brigitte Loenders, Xavier Charest-Morin, Jochen Knolle

**Affiliations:** ^1^Pharvaris Netherlands B.V., Leiden, Netherlands; ^2^AnalytiCon Discovery GmbH, Potsdam, Germany; ^3^Axe Microbiologie-Infectiologie et Immunologie, Research Center, CHU de Québec-Université Laval, Québec, QC, Canada

**Keywords:** bradykinin, B_2_ receptor, small molecule antagonists, icatibant, human umbilical vein

## Abstract

We here report the discovery and early characterization of Compound 3, a representative of a novel class of small molecule bradykinin (BK) B_2_ receptor antagonists, and its superior profile to the prior art B_2_ receptor antagonists Compound 1 and Compound 2. Compound 3, Compound 2, and Compound 1 are highly potent antagonists of the human recombinant B_2_ receptor (K_b_ values 0.24, 0.95, and 1.24 nM, respectively, calcium mobilization assay). Compound 3 is more potent than the prior art compounds and icatibant in this assay (K_b_ icatibant 2.81 nM). The compounds also potently inhibit BK-induced contraction of endogenous B_2_ receptors in a human isolated umbilical vein bioassay. The potencies of Compound 3, Compound 2, Compound 1, and icatibant are (pA_2_ values) 9.67, 9.02, 8.58, and 8.06 (i.e. 0.21, 0.95, 2.63, and 8.71 nM), respectively. Compound 3 and Compound 2 were further characterized. They inhibit BK-induced c-Fos signaling and internalization of recombinant human B_2_ receptors in HEK293 cells, and do not antagonize the venous effects mediated by other G protein-coupled receptors in the umbilical vein model, including the bradykinin B_1_ receptor. Antagonist potency of Compound 3 at cloned cynomolgus monkey, dog, rat, and mouse B_2_ receptors revealed species selectivity, with a high antagonist potency for human and monkey B_2_ receptors, but several hundred-fold lower potency for the other B_2_ receptors. The *in vitro* off-target profile of Compound 3 demonstrates a high degree of selectivity over a wide range of molecular targets, including the bradykinin B_1_ receptor. Compound 3 showed a lower intrinsic clearance in the microsomal stability assay than the prior art compounds. With an efflux ratio of 1.0 in the Caco-2 permeability assay Compound 3 is predicted to be not a substrate of efflux pumps. In conclusion, we discovered a novel chemical class of highly selective and very potent B_2_ receptor antagonists, as exemplified by Compound 3. The compound showed excellent absorption in the Caco-2 assay, predictive of good oral bioavailability, and favourable metabolic stability in liver microsomes. Compound 3 has provided a significant stepping stone towards the discovery of the orally bioavailable B_2_ antagonist PHA-022121, currently in phase 1 clinical development.

## Introduction

Bradykinin (BK)-related peptides, the kinins, are released from kininogens by kallikreins and exert various physiological and pathological effects *via* 2 related G protein-coupled receptors (GPCRs) termed the bradykinin B_1_ and B_2_ receptors (Leeb-Lundberg et al., 2005). While kinins and their receptors mediate compensatory and protective vasodilator effects under various pathological conditions, they are also mediators of inflammation, producing plasma extravasation and edema and pain (Leeb-Lundberg et al., 2005). Despite considerable efforts invested in antagonist drug development (Marceau and Regoli, 2004; Whalley et al., 2012), only one bradykinin receptor ligand is currently used in clinical practice, the B_2_ receptor antagonist icatibant, initially described decades ago (Hock et al., 1991). This synthetic peptide is short-lived and not orally bioavailable. When given subcutaneously, icatibant (Firazyr) aborts or limits attacks of hereditary angioedema (HAE) of type I and type II and attacks in patients with normal C1 inhibitor (HAE-nC1 INH) (Cicardi et al., 2014; Wu et al., 2016; Bouillet et al., 2016).

Many small molecule B_2_ receptor antagonists belonging to various chemotypes have been described (Leeb-Lundberg et al., 2005; Whalley et al., 2012). Two small molecules reached the clinic, but were injectables and discontinued due to lack of efficacy: anatibant (Shakur et al., 2009), for the treatment of traumatic brain injury and fasitibant for osteoarthritis (Tenti et al., 2016). The orally bioavailable B_2_ receptor antagonist FK 3657 was reported to be in clinical development but little is know about the results of the clinical studies and the fate of this compound (Abe et al., 2005). The feasibility to develop potent orally bioavailable B_2_ receptor antagonists was reported, but no clinical development candidate has been described from this series (Gibson et al., 2009).

The objective of the present work is to describe the key properties of a B_2_ receptor antagonist as a representative of a novel chemical class and how it compares to two related prior art compounds. We describe the *in vitro* pharmacology properties including: (1) *in vitro* antagonist potency *vs.* icatibant at the cloned recombinant human B_2_ receptor; (2) species specificity, as several B_2_ antagonists exhibit large potency differences as a function of the mammalian species (e.g., the bradyzide series; Marceau et al., 2003); (3) antagonist potency at the endogenous human B_2_ receptor according to the pA_2_ scale (Neubig et al., 2003) in the isolated umbilical vein, a standard model used to characterize B_2_ receptor ligands (Marceau et al., 1994; Marceau et al., 2003; Bawolak et al., 2007; Bawolak et al., 2008; Bawolak et al., 2009; Gera et al., 2016); (4) the competitive (surmountable) and reversible behavior in this model; (5) activity at the human bradykinin B_1_ receptor, itself antagonized by high concentrations of icatibant (Bastian et al., 1997), and at other receptor types represented in the umbilical vein; (6) The selectivity profile over a battery of more than 120 molecular targets. Furthermore we determined the permeability in a bidirectional Caco-2 assay and metabolic stability in a rat liver microsomes assay as first indicators of the drugability of the corresponding class of small molecule B_2_ receptor antagonists.

## Methods

### Materials

BK was purchased from Bachem Bioscience (Torrance, CA), icatibant, from Phoenix Pharmaceuticals (Burlingame, CA) and the B_1_ receptor agonist Sar-[D-Phe^8^]des-Arg^9^-BK, from Tocris Bioscience (Minneapolis, MN). In selectivity experiments, serotonin creatinine sulfate (Millipore-Sigma) and U-46619 (Enzo Life Sciences, Farmingdale, NY) were also exploited.

**Figure 1:**
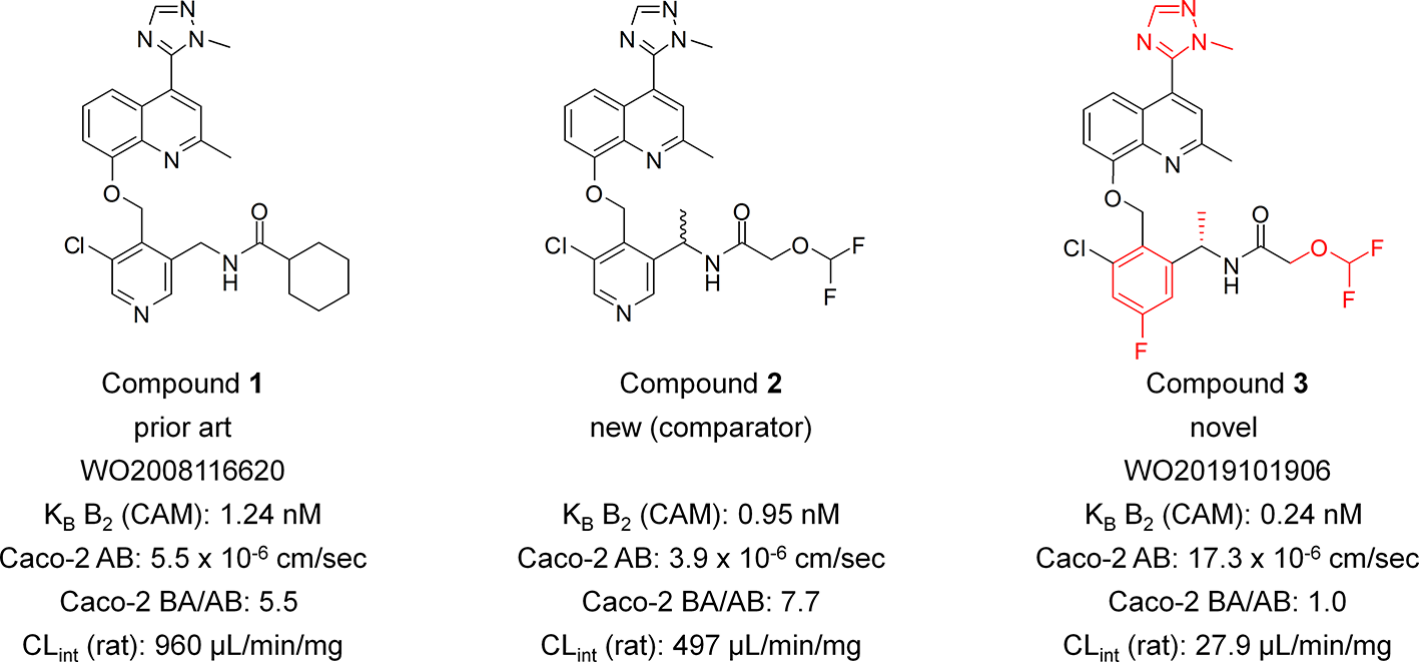
Structures of Compound 1, Compound 2, and Compound 3 and their selected key properties. Key structural elements of the novel series indicated in red. Caco-2 AB, permeation rate apical to basolateral direction. Caco-2 BA/AB, ratio of permeation rates basolateral to apical direction and apical to basolateral direction.

Compound 1: *N*-((5-chloro-4-((2-methyl-4-(1-methyl-1*H*-1,2,4-triazol-5-yl)quinolin-8-yloxy)methyl)pyridin-3-yl)methyl)cyclohexanecarboxamide; Compound 2: *rac*-*N*-(1-(5-chloro-4-((2-methyl-4-(1-methyl-1*H*-1,2,4-triazol-5-yl)quinolin-8-yloxy)methyl)pyridin-3-yl)ethyl)-2-(difluoromethoxy)acetamide; Compound 3: (*S*)-*N*-(1-(3-chloro-5-fluoro-2-((2-methyl-4-(1-methyl-1*H*-1,2,4-triazol-5-yl)quinolin-8-yloxy)methyl)phenyl)ethyl)-2-(difluoromethoxy)acetamide. (**Figure 1**). The compounds have been synthesized as described previously (Gibson et al., 2019). For detailed experimental procedures for the synthesis of Compound 3 we refer to example 6 in the patent (preparation of Compound no. 6). Purities by HPLC (215 nm) for all three compounds was >99%, the supplier of the compounds was AnalytiCon Discovery GmbH.

### Competition Binding Assay

The ability of the test items to inhibit [^3^H]BK binding to the human B_2_ receptors was determined in human recombinant CHO cells expressing the cloned human B_2_ receptor. Cell membrane homogenates were incubated for 60 min at 22°C with 0.3 nM [^3^H]BK (Kd value 0.32 nM) in the absence or presence of the test compound in a buffer containing 50 mM Tris/HCl (pH 7.4), 0.2 g/L 1-10-phenanthroline and 0.1% BSA. Nonspecific binding is determined in the presence of 1 µM BK. Following incubation, the samples are filtered rapidly under vacuum through glass fiber filters presoaked with 0.3% PEI and rinsed several times with ice-cold 50 mM Tris-HCl using a 96-sample cell harvester (Packard). The filters are dried then counted for radioactivity in a scintillation counter (Topcount, Packard) using a scintillation cocktail. The results are expressed as percent inhibition of the control radioligand specific binding. The standard reference compound used is the B_2_ antagonist NPC 567 (Bachem), which was tested in each experiment at several concentrations to obtain a competition curve from which its IC_50_ was calculated. Test items and icatibant were evaluated at final concentrations between 10^-11^ and 10^-6^ M.

### Ca^2+^ Mobilization Assay

#### Human B_2_ Receptor

The agonist and antagonist potency of the test items at the human B_2_ receptor were evaluated in transfected CHO cells by measuring their direct effect (agonism) and their inhibitory effect of BK-induced cytosolic Ca^2+^ mobilization using a fluorimetric detection method, according to the methods established at Eurofins (Celle l'Evescault, France). The cells were suspended in DMEM buffer, then distributed in microplates. The fluorescent probe (Fluo4 Direct, Invitrogen) was mixed with probenecid in HBSS buffer complemented with 20 mM Hepes (pH 7.4) and was added to each well and equilibrated with the cells for 60 min at 37°C, then 15 min at 22°C. First, for the measurement of agonist potency, test compound and reference agonist BK were added to the cells at several concentrations to generate a concentration-response curve, and the change in fluorescence intensity was measured. The results are expressed as a percent of the control response to 3 nM BK. Where possible, EC_50_ values of test items were calculated. Second, the antagonist activity of the compounds was evaluated by measuring their effect on BK-induced cytosolic Ca^2+^ mobilization. For this, test compound, reference antagonist icatibant or HBSS buffer were added to the cells at several concentrations to generate concentration–response curves, left for 5 min, then 0.03 nM BK was added and the changes in free cytosolic Ca^2+^ concentration was measured as a change in fluorescence intensity. The EC_50_ value of BK in this assay was 6.05 ± 2.86 pM (mean ± S.D.). The results are expressed as a percent inhibition of the control response to 0.03 nM BK. IC_50_ values were calculated where possible. Test items and icatibant were evaluated at final concentrations between 10^-11^ and 10^-6^ M.

#### Cynomolgus B_2_ Receptor

HEK293 cells expressing the B_2_ receptor of cynomolgus monkeys (*Macaca fascicularis*) were constructed in the laboratories of Prof. Alexander Faußner, Ludwig-Maximilians-Universität München. The B_2_ receptor gene was introduced *via* a pcDNA5/FRT derived plasmid. The cells are cultured in DMEM medium supplemented with 10% heat-inactivated fetal calf serum, 2 mM L-glutamine, 200 units/mL of potassium penicillin and 100 µg/mL of streptomycin sulfate, 10 µg/mL blasticidin and 250 µg/mL hygromycin. Experiments were conducted at AnalytiCon (Potsdam, Germany) according to their established methods and conditions. The cells were suspended in culture medium with supplemental fetal calf serum reduced to 5% into 96 well plates coated with 0.2 mg/L PDL at an appropriate density. The cells were loaded with a Ca^2+^- dependent fluorescent dye (FLIPR^®^ Calcium 6 Assay Kit). For the intrinsic agonist response, test compound was added to the cells using FlexStation 3 (Molecular Devices, LLC) and change of fluorescence was recorded. Test item was then left on the cells for 30 min, and BK was added at its EC_80_ concentration to measure antagonism of BK, recorded as a change in fluorescence of the calcium sensitive dye (excitation 485 nM, emission 525 nm). The EC_80_ concentration of BK used, was determined in the morning of each experiment and ranged between 365 and 710 pM. The average EC_50_ value of BK was 155 ± 43 pM, the average EC_80_ value was 539 ± 160 pM (mean ± S.D.). Compound 3 and icatibant were tested between 10^-11^ and 10^-6^ M.

#### Dog, Rat, And Mouse B_2_ Receptor

The determination of antagonist potencies of test items to inhibit BK activation of rat, mouse and dog B_2_ receptors was conducted by Multispan Inc (Hayward, California USA). HEK293T cells, stably expressing the recombinant rat, mouse, and dog bradykinin B_2_ receptor, were grown in DMEM, 10% FBS, 1 μg/mL puromycin. On the day of assay, cells were seeded in 384-well plate into HBSS assay buffer at an appropriate density. The calcium assay was conducted on the same day. For this, the calcium dye loading buffer (Multispan Inc., Cat# MSCA01-1), containing Fluo-8 loading dye and probenecid, was added to the cells and incubated for 30 min at 37°C and 30 min at room temperature. Cells were preincubated with Compound 3 for 30 min at room temperature prior to the addition of BK. Ca^2+^- flux was monitored when test compound was added to the wells, and again 30 min later, when the agonist BK is added at the EC_80_ concentration (4.32, 0.53 and 1.86 nM for rat, mouse and dog B2 receptor, respectively). Excitation/emission wavelengths were 490 and 525 nm respectively. Compound 3 was tested between 10^-10^ and 10^-5^ M, icatibant was tested between 10^-15^ and 10^-6^ M.

### Profiling Across a Panel of Molecular Targets

The binding affinity or functional activity of Compound 3 was measured for a range of 138 molecular targets, including receptors, ion channels, enzymes and transporters, according to methods established by Eurofins (Celle l'Evescault, France). Assay conditions are summarized in **Table S2** (Supplementary material).

### Antibody Internalization Assay

B_2_ receptor-mediated transport of immune complexes constructed at the cell surface was previously reported (Bawolak et al., 2011). Intact HEK 293a cells stably expressing the *N*-terminally myc-tagged B_2_ receptor construction (human sequence, Charest-Morin et al., 2015) were treated with the monoclonal anti-myc antibody (clone 4A6) conjugated with AlexaFluor-488 (Millipore, dilution 1:500) added to the culture medium. Cells were further incubated at 37°C. Without rinsing, a selected antagonist or their DMSO vehicle was added 5 min later, and BK (100 nM) or its saline vehicle 15 min later. After an additional 30 min incubation period at 37°C, cells were rinsed 3 times with Hank's balanced salt solution (Multicell Wisent, St. Bruno, Canada) and photographed in transmission and epifluorescence using an Olympus BX51 microscope coupled to a CoolSnap HQ digital camera (filters for AlexaFluor 488: excitation 460–500 nm, emission 510–560 nm).

### Human Umbilical Vein Pharmacology

The local ethics committee (CHU de Québec-Université Laval) had approved the project (File no. 2017-3720). Umbilical cords were obtained following elective caesarean sections; all problematic deliveries are excluded. Informed consent was obtained from the mothers. The cords were transported from the maternity ward to the laboratory in sterile Hank's balanced salt solution containing penicillin and streptomycin. Segments of umbilical veins were dissected carefully from the cords and a metal rod was inserted in the lumen. Excess connective tissue was excised and rings (2–3 mm wide) were cut. Rings of umbilical veins were suspended in 5-mL organ chambers containing oxygenated (95% O_2_, 5% CO_2_) and warmed (37°C) Krebs' solution between a metal hook and a thread loop. This solution had the following composition: NaCl 117.5 mM, KCl 4.7 mM, KH_2_PO_4_ 1.2 mM, MgSO_4_ 1.18 mM, CaCI_2_ 2.5 mM, NaHCO_3_ 25.0 mM, and D-glucose 5.5 mM. A baseline of one gram-weight was applied to these preparations. Changes of tension were recorded by using isometric transducers (model 52-9545, Harvard Bioscience, South Natick MA). Eight tissue baths are operated in parallel.

For the evaluation of drugs as B_2_ receptor antagonists, the tissues were equilibrated for 3 h before starting the experiments. The full cumulative concentration-effect curve of synthetic BK (1 to 10,000 ng/mL, optionally 25,000 ng/mL) was constructed with 7 or 8 concentrations of BK in the presence or absence of the tested antagonist drug or its vehicle (saline or DMSO, not exceeding 0.1% v/v final concentration for DMSO). The antagonist drugs were applied to the bathing fluid 30 min before the construction of the curve. The usual design was to test the vehicle control and 4 concentrations of the antagonist (e.g., 1, 10, 100 nM, 1 μM, and possibly 10 μM). The set of 8 tissues can be reused at time 5 h with ample washing with fresh Krebs, allowing the testing of the same or larger antagonist concentrations in each tissue bath. The maximal response to BK can be estimated from the first concentration-effect curve if a high concentration of antagonist (usually tested at time 5 h) is believed to depress the maximal response at the maximal cumulative BK concentration tested, 34.1 μM.

We evaluated the selectivity of Compound 2, Compound 3 and icatibant for B_2_ receptors *versus* other endogenously expressed receptors in the human umbilical vein. For this purpose, contractile responses to serotonin, acting on the 5-HT_2A_ receptor, and U-46619, acting at the prostanoid TP receptor, were measured (Rogines-Velo et al., 2002; Daray et al., 2003). The experiments were conducted in the presence or absence of Compound 2, Compound 3 or icatibant, applied 30 min before the agonist.

Compound 3 and icatibant were also tested against an agonist of the kinin B_1_ receptor, Sar-[D-Phe^8^]des-Arg^9^-BK. This highly selective agonist of the B_1_ receptor is resistant to multiple peptidases and adapted to the umbilical vein preparation (Houle et al., 2000; Jean et al., 2016). In this special procedure, tissues were incubated with interleukin-1β (5 ng/mL) and tumor necrosis factor-α (10 ng/mL) during the 3-hour incubation period to upregulate the expression of B_1_, and then amply washed (Jean et al., 2016). The cumulative concentration-effect of Sar-[D-Phe^8^]des-Arg^9^-BK was constructed 4 h after tissue mounting and antagonists were applied 30 min before this.

#### Data Analysis for Umbilical Vein Pharmacology

Each curve allows the determination of a maximal effect (expressed in % maximal BK effect for competitive antagonists) and sensitivity (EC_50_) of each preparation to BK. Prism 5.0 (GraphPad) was used to draw concentration–effect curves (least square fitting of sigmoidal dose–response equation with variable slope) and to derive contractile EC_50_ values. The effect of each antagonist drug at each tested concentration was calculated as the rightward shift (dose ratio, DR) of the averaged concentration–effect curve relative to the EC_50_ established in control tissues from the same donor exposed only to the vehicle of the drugs. When applicable, Schild plot parameters (pA_2_ ± S.E.M., slope ± S.E.M.) were estimated from the Schild plot (abscissa: -log[antagonist], ordinate: log(DR-1), linear regression) using the computer program Pharm/PCS (Tallarida and Murray, 1987). For this calculation, each point of the regression was calculated using data from each separate day of experiments because day-to-day contingencies, that include the identity of tissue donor, were felt to be the most important source of variability. This computerized procedure also calculated the slope of the regression ± S.E.M. that should be close to −1 for competitive agonists. Two-way ANOVA has been applied to the data used for generating the pA_2_ values. In this analysis, the 2 independent categories are the antagonist drug identity (4 drugs) and concentration (only the range common to all 4 antagonists: 10, 100, and 1000 nM). The numerical variable was log(DR-1) (data sets compatible with normal distribution and homogenous variances). The Bonferroni post-hoc test was used to compare the effect of the drugs at each concentration (Prism 5.0).

### Caco-2 Permeation Assay

The permeability of the test compounds was determined at 10 µM across monolayers formed by the human colon carcinoma cell line Caco-2 according to standard operating procedures at Pharmacelsus (Saarbruecken, Germany). Caco-2 cell cultures (ACC 169, Leibniz Institut DSMZ GmbH) were maintained and subcultured at 37°C, 5% CO_2_ and 95% humidity. For the assays, the cells were seeded at a concentration of 1.5 x 10^4^ cells/well on permeable membrane inserts with a growth surface of 1.1 cm^2^ (Transwell^®^ plates). The cells were cultured for 21 days and the medium was renewed 3 times a week. Once a week the cell monolayer was quality controlled. The bidirectional Caco-2 assay was performed as described by Hubatsch et al., 2007, with minor modifications. Prior to assay start, the cells were washed twice with HBSS and then the monolayer was equilibrated in pre-warmed apical and basolateral buffer for 20 min at 37°C. The assay was started by replacing the apical or basolateral buffer by the test or reference item solutions depending on the permeation direction. During the experiment, the Transwell^®^ plates were incubated at 37°C at 20 rpm in a shaking water bath. Samples of 200 µl were taken from the basolateral and apical side, respectively, after 60 min and were replaced by drug-free basolateral or apical buffer. In order to assess the mass balance (recovery) of the experiments, a sample from each basolateral compartment was taken after an additional 60 min. Samples were processed for ACN precipitation and analysis by LC/MS.

The apparent permeation coefficient (Papp) was calculated using the following equation:

Equation 1: Papp [cm/sec] = ΔQ/Δt *1/A *1/Co

ΔQ/Δt = diffusion rate of compound [μg/s] (i.e. slope of concentration vs. time relation)
A = total membrane surface [cm^2^]
Co = initial concentration [μg/mL]

To consider the efflux by P-glycoprotein and other transporters, the ratio of both transport directions was calculated:

Equation 2: Efflux = Papp (B-A) / Papp (A-B)

Papp B-A = apparent permeation from basolateral chamber to apical chamber
Papp A-B = apparent permeation from apical chamber to basolateral chamber

### Microsomal Stability

The metabolic stability of the test compounds was determined according to Obach, 1999. Assays were performed in 96-deepwell-plates using liver microsomes pooled from male Wistar rats (Corning). Rat liver microsomal incubations were conducted in duplicate and incubation mixtures consisted of liver microsomes (0.5 mg microsomal protein/mL), test compound (1 µM), MgCl_2_ (2 mM), and NADPH (1 mM) in a total volume of 0.7 mL sodium phosphate buffer (100 mM, pH 7.4). Reactions were commenced with the addition of NADPH and shaken on a horizontal shaker with a fitted heating block at 37°C. At t = 0 min and the time points 10 min, 30 min and 60 min, aliquots (70 µL) were removed from the incubations and added to 140 µL termination mixtures. Termination mixtures consisted of acetonitrile supplemented with diazepam, diclofenac and griseofulvin as internal analytical standards. The quenched samples were processed by mixing and centrifugation (2,200 x g, 5 min). The particle free supernatant was diluted (1:1, v/v) with deionized water and subsequently subjected to LC-MS for quantitative bioanalysis in terms of test compound depletion (pump flow rate: 600 µL/min; Kinetex Phenyl-Hexyl analytical column 2.6 µm, 50x2.1 mm (Phenomenex, Germany)). Incubations containing verapamil at a concentration of 1 µM were used as high clearance positive control (PC; n=2), and incubations without NADPH (70 µL phosphate buffer (supplemented with 2 mM MgCl_2_) instead of 70 µL NADPH solution), in order to verify that any apparent loss of test item in the assay incubation was due to metabolism, were used as negative control (NC; n=2). The rate of disappearance of a test compound over time is measured and used to calculate *in vitro* intrinsic clearance (CL_int_).

### Data Analysis

Numerical values are reported as means ± standard deviation (S.D.) or standard error of the mean (S.E.M.), as indicated for each assay. Sets of values were compared with ANOVA followed by Dunnett's or Tukey's post-hoc test (Prism 5.0).

## Results

### Binding to Recombinant Human B_2_ Receptors

[^3^H]BK had previously shown saturable binding to the membrane fraction of the CHO cells stably expressing recombinant human B_2_ receptors with an apparent K_d_ of 0.32 nM, in line with the reported affinity of [^3^H]BK (0.45 nM) for the recombinant human B_2_ receptor (Pruneau et al., 1998). NPC 567, the internal reference antagonist showed a K_i_ value in our experiments of 7.2 nM also in line with its reported affinity (4.1 nM) for the cloned human B_2_ receptor (Paquet et al., 1999).

All compounds fully inhibited 0.3 nM [^3^H]BK binding to the recombinant human B_2_ receptors. IC_50_ and K_i_ values are summarized in **Table 1**. Compound 3 and icatibant exhibited subnanomolar affinity for the human B_2_ receptor (K_i_ values 0.50 and 0.60 nM, respectively). The affinities of Compound 1 and Compound 2 were lower, but still in the low nanomolar range (K_i_ values 3.8 and 3.9 nM, respectively).

**Table 1:** Affinities, IC_50_ and K_i_ values at the human B_2_ receptor derived from the [^3^H]BK binding competition assay.

	IC_50_ (nM)			K_i_ (nM)			n
	Mean	S.D.	Geometric mean	Mean	S.D.	Geometric mean	
Icatibant	1.21	0.46	1.15	0.64	0.26	0.60	6
Compound 1	7.4	–	7.4	3.8	–	3.8	1
Compound 2	7.6	–	7.6	3.9	–	3.9	1
Compound 3	0.97	0.03	0.97	0.50	0.02	0.50	3

### Antagonism of Recombinant Human B_2_ Receptors

Administration of BK to CHO cells stably expressing recombinant human B_2_ receptors, elicited Ca^2+^- ion mobilization with an average EC_50_ value of 6 pM, in line with the reported BK agonist potency of 9 pM at the cloned human B_2_ receptor in CHO cells (Zhang et al., 2001).

**Table 2** summarizes the potencies of Compound 1, Compound 2, Compound 3, and icatibant to inhibit BK activation of recombinant human B_2_ receptors expressed in CHO cells, using a calcium mobilization assay.

**Table 2:** Antagonist potencies, IC_50_ and K_b_ values at the human B_2_ receptor in the calcium mobilization assay.

	IC_50_ (nM)			K_b_ (nM)			n
	Mean	S.D.	Geometric mean	Mean	S.D.	Geometric mean	
Icatibant	27.8	7.6	25	3.19	1.47	2.81	13
Compound 1	11.3	4.1	11	1.30	0.46	1.24	3
Compound 2	9.57	6.46	8.3	1.08*	0.71	0.95	3
Compound 3	2.13	0.12	2.13	0.24**	0.02	0.24	3

ANOVA, P < 0.01. *P < 0.05; **P < 0.01 for comparison with the common control value, icatibant K_b_ (Dunnett's test).

Compound 1, Compound 2 and Compound 3 all demonstrated high antagonist potency towards the human B_2_ receptor. Compounds 2 and 3 were significantly more potent as compared to icatibant with Compound 3 being the most active compound. The K_b_ values were 1.24, 0.95 and 0.24 nM, respectively, versus 2.81 nM for icatibant.

### Species Selectivity

As the bradykinin B_2_ receptor is well known for its species selective pharmacology (Paquet et al., 1999; Burgess et al., 2000), we investigated the antagonist potency of Compound 3 in cells expressing B_2_ receptors from human, cynomolgus monkey, dog, rat, and mouse (**Table 3**). The K_b_ value of Compound 3 to antagonize BK activation of the cynomolgus monkey B_2_ receptor was found to be in the same range as for the human B_2_ receptor (1.36 nM versus 0.24 nM). Compound 3 was however more than 500-fold less potent in antagonizing the rat, and mouse B_2_ receptor, and more than 10,000-fold less potent towards the dog B_2_ receptor. Icatibant in contrast, showed a more or less stable antagonist potency across species (**Table 3**).

**Table 3:** Antagonist potencies of Compound 3 and icatibant at the B_2_ receptor from different species.

	Compound 3					Icatibant				
	IC_50_ (nM)		K_b_ (nM)		n	IC_50_ (nM)		K_b_ (nM)		n
	Mean	S.D.	Mean	S.D.		Mean	S.D.	Mean	S.D.	
Human	2.13	0.12	0.24	0.02	3	27.8	13.4	3.19	1.55	16
Monkey	6.25	2.11	1.36	0.34	5	17.1	5.64	4.06	0.99	5
Dog	~7,600	NA	~2,700	–	1	87.1	NA	30.7	–	1
Rat	1,034	329	147	60	3	37.4	6.4	5.60	2.20	3
Mouse	~1,200	NA	~350	–	1	15.5	NA	4.4	–	1

### Pharmacology in the Human Umbilical Vein

**Figure 2:**
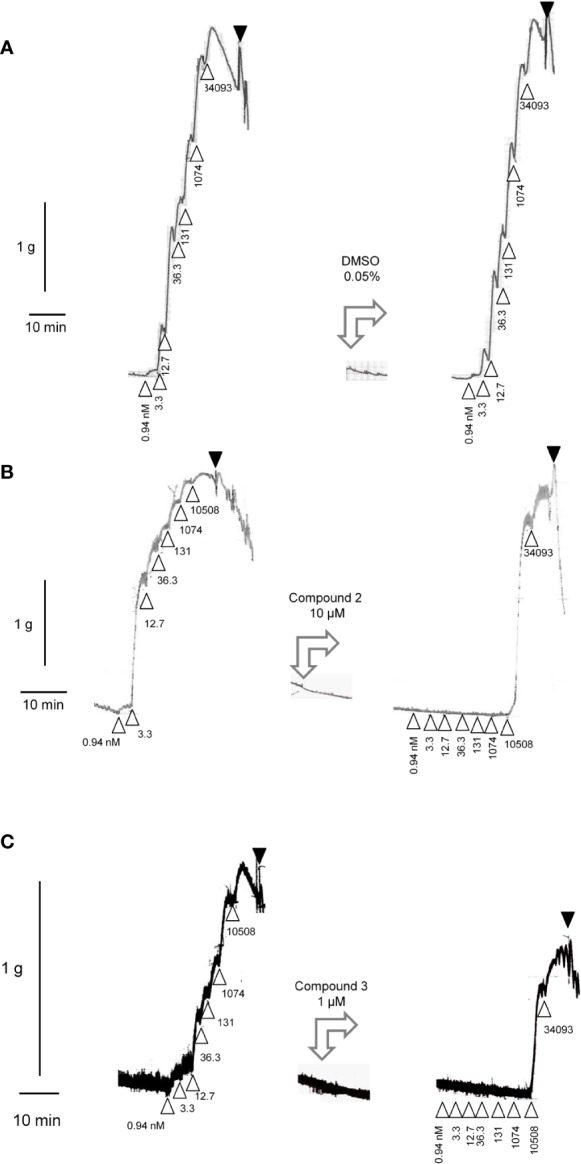
All panels, left tracings: Construction of a cumulative concentration-effect curve for BK-induced contraction by using a ring of human umbilical vein pre-equilibrated for 3 h. Abscissa scale: time; ordinate scale: isometric contraction, grams. Δ indicates the application of BK (cumulative nanomolar concentration indicated) and ▼ the first of a series of washouts. All panels, middle tracings: the tested small molecule antagonists or their DMSO vehicle exerted no contractile effects. All panels, right tracings are recorded in the same tissues at time 5 h. The DMSO vehicle has no major effect on the previously recorded response to BK (sensitivity, maximal effect) **(A)**. Compound 2 **(B)** or Compound 3 **(C)** at a high concentration depressed both the sensitivity of the same ring to BK and, slightly, the maximal effect of the peptide (estimated from the previously recorded E_max_).

**Figure 3:**
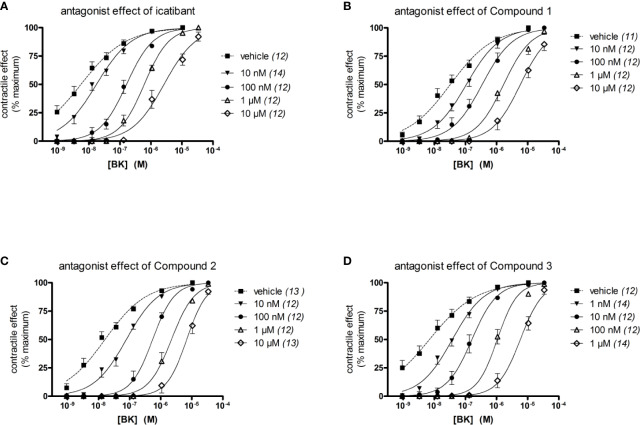
Effect of four B_2_ receptor antagonists on BK-induced contraction in the human isolated umbilical vein. The identity of the tested antagonist is icatibant **(A)**, Compound 1 **(B)**, Compound 2 **(C)** and Compound 3 **(D**). Values are the means ± S.E.M. of the number determinations indicated between parentheses. For the sake of clarity, several error bars below or above the average values have been omitted.

**Figure 4:**
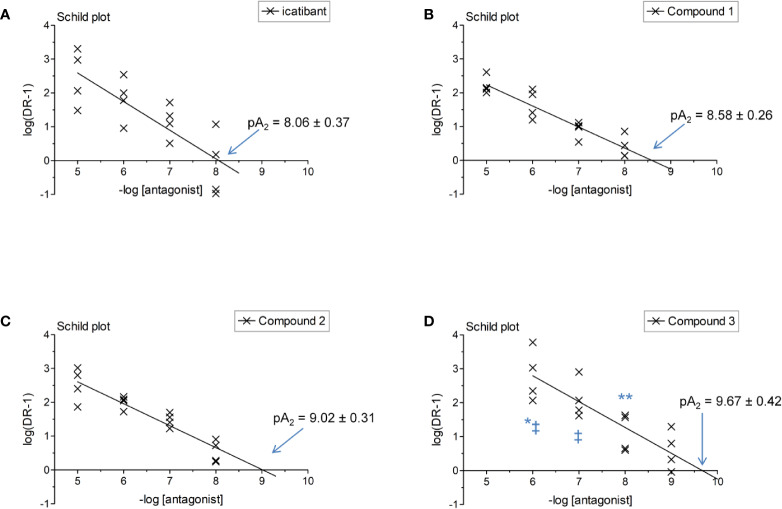
Schild plot analysis of **Figure 3** data where a point in the regression represents one day of experimental data for a given antagonist concentration. The identity of the tested antagonist is icatibant **(A)**, Compound 1 **(B)**, Compound 2 **(C)** and Compound 3 **(D)**. Two-way ANOVA indicated that the antagonist drug identity and concentration determined the variation of log(DR-1) values (P < 10^-4^ for each). Values different from those of icatibant: *P < 0.05; **P < 0.01; values different from those of Compound 1: ^‡^P < 0.05 (Bonferroni post-hoc test).

The construction of a cumulative concentration-effect curve for BK-induced contraction of the umbilical vein in the absence or presence of an antagonist or the DMSO vehicle is graphically illustrated in **Figure 2** and further explained in **Figure 2** legend. None of the four B_2_ receptor antagonists tested showed any intrinsic partial agonist effect when applied to human umbilical vein rings (illustrated for Compound 3 and Compound 2 in **Figure 2**). The clinically used peptide antagonist icatibant was studied in the umbilical vein contractility assay for comparison with the small molecule compounds; its vehicle was 0.9% saline. Icatibant behaved as a competitive (surmountable) antagonist of BK in the isolated human umbilical vein preparation (**Figure 3A**). This can be further described as a dose-dependent rightward shift of the BK concentration-effect curve with no depression of the maximal effect. Further evidence of a lack of effect on BK contractile E_max_ is provided in **Table S1** (Supplementary material): when expressed in force units (gram-weight), the maximal BK-induced contractions recorded in the absence or presence of various icatibant concentrations were similar. **Figure 4A** shows a Schild plot where each experimental point in the regression represents the log(DR-1) value for each experimental day, as the tissue donor identity is felt to be a major determinant of variability. Using this method applied by the computer program Pharm/PCS, the pA_2_ value for icatibant is 8.06 ± 0.37, comparable to the previously reported value of 8.2 (Marceau et al., 1994), and the slope of the regression, -0.85 ± 0.16 (**Table 4**), compatible with competitive antagonism.

**Table 4:** pA_2_ value estimates for compounds tested as B_2_ receptor antagonists using the umbilical vein contractility assay.

Compound	Parameters calculated using Pharm/PCS (**Figure 4**)			
	pA_2_	pA_2_ S.E.M.	Slope	Slope S.E.M.
icatibant	8.06	0.37	−0.85	0.16
Compound 1	8.58	0.26	−0.62	0.07
Compound 2	9.02	0.31	−0.65	0.07
Compound 3	9.67	0.42	−0.76	0.13

The vehicle for the three small molecule antagonists was DMSO 0.05% v/v. The compounds were more potent than icatibant in the following order: Compound 1 (pA_2_ 8.58), Compound 2 (9.02) and Compound 3 (9.67) (**Figures 3B–D** and **4B–D** and **Table 4**). Thus, Compound 3 showed a picomolar potency in this model (0.21 nM), approximately 40-fold more potent than icatibant (8.7 nM). Two-way analysis of variance of **Figure 4** data showed that the significant sources of variation for log(DR-1) values were the antagonist drug identity (P < 10^-4^) and concentration (P < 10^-4^). Further, the Bonferroni post-hoc test evidenced that Compound 3 was more potent than either icatibant or Compound 1 at specific concentrations (**Figure 4**). The small molecule antagonists were surmountable, based on lack of effect on BK contractile E_max_ (**Figure 3**, **Table S1**), but the Schild plot slopes were somewhat inferior to 1 (**Table 4**), possibly indicating a not fully competitive behavior.

**Figure 5:**
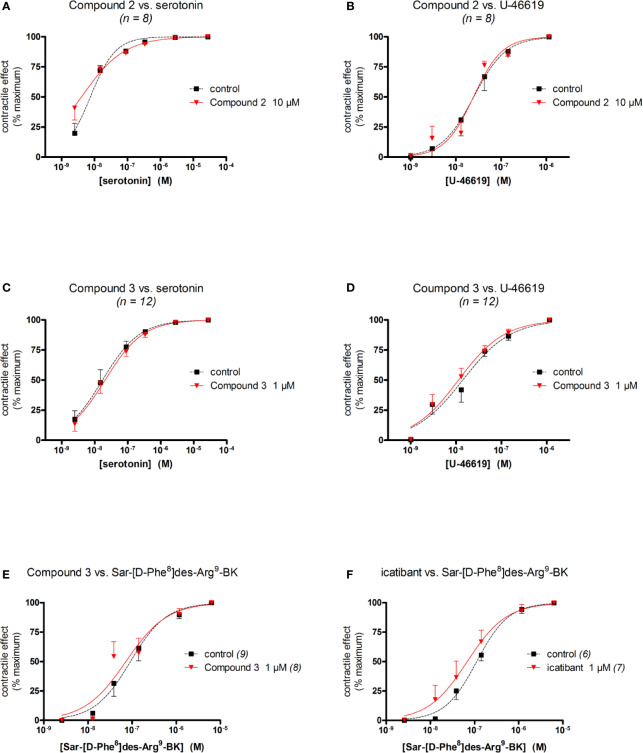
Specificity of the antagonist effect of Compound 2 **(A, B)**, Compound 3 **(C–E)** or icatibant **(F)** in the human umbilical vein preparation (concentrations as indicated, replicate number indicated by *n*). **(A, C)** Lack of antagonist effect on serotonin-induced contraction. **(B, D)** Lack of antagonist effect on U-46619-induced contraction. **(E, F)** Lack of Compound 3 and icatibant on the B_1_ receptor agonist Sar-[D-Phe^8^]des-Arg^9^-BK-induced contraction. Values are the means ± S.E.M. of the number of determinations indicated by *n*. For the sake of clarity, several error bars below or above the average values have been omitted. Agonist EC_50_ values and overlapping confidence limits are reported in **Table 5**.

The umbilical vein contractile assay also allows to assess other GPCRs (Altura et al., 1972; Gobeil et al., 1996). We performed additional experiments to characterize the specificity of the most potent antagonists, Compound 2 and Compound 3 (**Figure 5**, **Table 5**). Compound 2, at 10 μM, failed to modify the serotonin (5-hydroxytryptamine)-mediated concentration–effect curve which is predominantly mediated by 5-HT_2A_ receptors (Rogines-Velo et al., 2002; **Figure 5A**) and failed to modify the thromboxane A_2_ mimetic U-46619-induced contractile response, which activates the TP prostanoid receptors in the umbilical vein (Daray et al., 2003; **Figure 5B**). The same conclusions applied to Compound 3 tested at 1 μM against serotonin and U-46619 (**Figures 5C, D**). Both Compound 3 and icatibant, at 1 μM, failed to antagonize the contractile effect of the B_1_ receptor agonist Sar-[D-Phe^8^]des-Arg^9^-BK in the umbilical vein (**Figures 5E, F**). EC_50_ values of each agonist in the presence or absence of each antagonist are reported in **Table 5** along with overlapping 95% confidence limits on the values.

**Table 5:** Agonist EC_50_ values in specificity experiments (**Figure 5**).

Antagonist	Agonist	Antagonist concentration	Agonist EC_50_ (nM)	95% confidence limits on EC_50_ values
Compound 2	Serotonin	0	7.5	5.9 – 9.5
		10 μM	4.0	2.6 – 6.1
	U-46619	0	24.9	17.2 – 36.2
		10 μM	24.5	18.3 – 32.9
Compound 3	Serotonin	0	17.2	11.5 – 25.8
		1 μM	20.5	14.5 – 28.9
	U-46619	0	14.1	9.7 – 20.5
		1 μM	11.3	8.8 – 15.5
	Sar-[D-Phe^8^]des-Arg^9^-BK	0	92.0	62.9 – 134.7
		1 μM	72.3	43.9 – 119.2
icatibant	Sar-[D-Phe^8^]des-Arg^9^-BK	0	112.1	92.2 – 139.3
		1 μM	68.2	42.5 – 109.4

**Figure 6:**
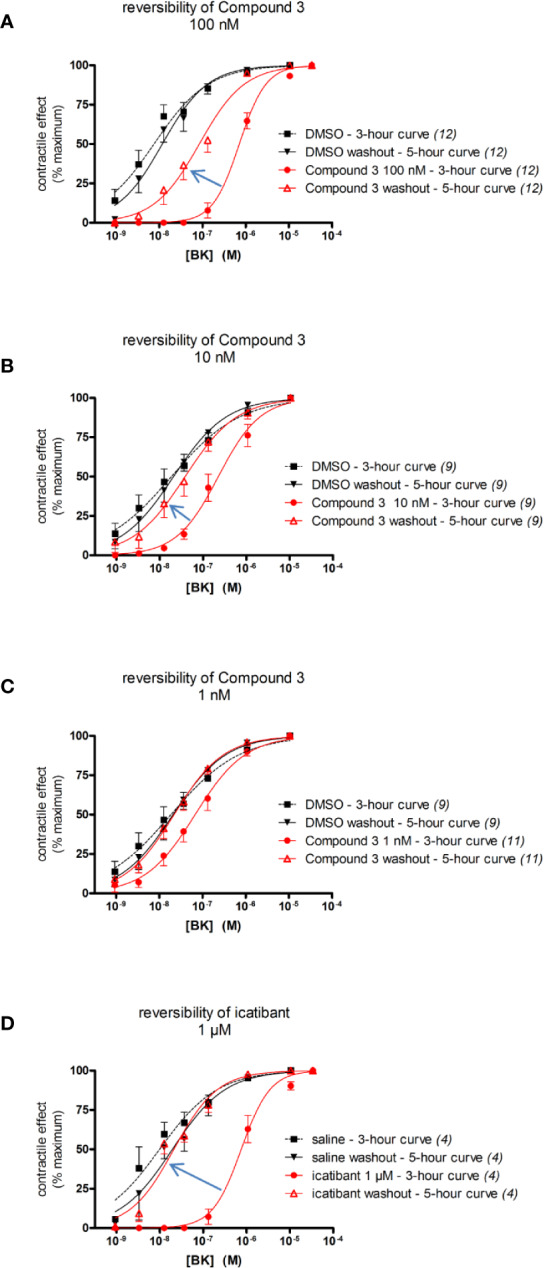
Reversibility of the antagonist effect of selected B_2_ receptor antagonists (concentrations as indicated, replicate number indicated between parentheses) in the human umbilical vein preparation following ample washing with fresh Kreb's solution. The apparent BK EC_50_ values are reported in **Tables 6** and **7**. **(A–C)** Reversibility of Compound 3 (100 nM, 10 nM, and 1 nM, respectively). **(D)** Reversibility of icatibant, 1 μM. The experiments reported in **(B)** and **(C)** have been conducted in parallel and share the same DMSO controls (presented twice in these panels). Values are the means ± S.E.M. of the number of determinations indicated by *n*. For the sake of clarity, several error bars below or above the average values have been omitted.

In other experiments, the reversibility of the most potent antagonist, Compound 3, has been tested (**Figure 6**). A 100 nM concentration, that strongly antagonizes BK without depressing its maximal effect, has been applied 30 min before the initial construction of the BK concentration effect curve (performed at 3 h after tissue mounting). At the end of the BK concentration curve, the antagonism was fully overcome by high BK concentrations, suggesting full displacement. Then the tissues were amply washed for about 100 min. The BK curve construction was repeated (at about 5 h after tissue mounting) to assess reversibility (**Figure 6A**). The potency of BK after Compound 3 antagonism and washout, was about 7.2-fold lower as compared to the potency of BK in control tissue tested at 5 h, indicating partial or slow reversibility (**Table 6**). In contrast, the EC_50_ of BK remained approximately constant from the 3 hour to the 5 hour curves following treatment with 1 μM icatibant, a concentration that produced approximately the same rightward shift intensity as that selected for 100 nM Compound 3 (**Figure 6D**, BK EC_50_ values and 95% confidence limits of these experiments reported in **Tables 6** and **7**). The effects of the more therapeutically relevant Compound 3 concentrations of 1 and 10 nM were fully reversible when this protocol was applied (**Figures 6B, C** and **Table 6**).

**Table 6:** Apparent BK EC_50_ values in the Compound 3 reversibility experiment (**Figures 6A–C**).

Compound 3 concentration	Curve, time point	BK EC_50_ (nM)	95% confidence limits on EC_50_ values
100 nM (**Figure 6A**)	Vehicle, 3 h	7.5	5.1 – 11.1
	Vehicle washout, 5 h	11.7	8.3 – 16.5
	Compound 3 100 nM, 3 h	713	613 – 829
	Compound 3 washout, 5 h	84.1	57.9 – 122.2
10 nM (**Figure 6B**)	Vehicle, 3 h	18.7	11.8 – 29.4
	Vehicle washout, 5 h	20.8	15.0 – 28.7
	Compound 3 10 nM, 3 h	224.9	164.5 – 307.4
	Compound 3 washout, 5 h	38.5	25.1 – 59.1
1 nM (**Figure 6C**)	Vehicle, 3 h	18.7	11.8 – 29.4
	Vehicle washout, 5 h	20.8	15.0 – 28.7
	Compound 3 1 nM, 3 h	67.4	47.9 – 94.9
	Compound 3 washout, 5 h	22.0	16.8 – 28.6

**Table 7:** Apparent BK EC_50_ values in the icatibant reversibility experiment (**Figure 6D**).

Curve, time point	BK EC_50_ (nM)	95% confidence limits on EC_50_ values
Vehicle, 3 h	9.9	6.0 – 16.2
Vehicle washout, 5 h	19.5	11.8 – 32.5
icatibant 1 μM, 3 h	756	599 – 954
icatibant washout, 5 h	21.1	15.9 – 27.9

### Effect of Compound 2 and Compound 3 on BK-Induced Signaling in HEK 293a Cells

c-Fos accumulation is a distal response to the stimulation of numerous receptors, including the bradykinin B_2_ receptor (Bawolak et al., 2009). Over 60 min, 10 nM BK induced c-Fos accumulation in HEK 293a that stably expressed human myc-B_2_ receptor, but not in untransfected cells (**Figure S1**, Supplementary material). Co-treatment with Compound 2 or Compound 3 inhibited this response in a concentration-dependent manner (between 10 nM and 1 μM). The 10-fold excess of the former antagonist (100 nM) significantly reduced the response to BK (10 nM) whereas the latter had a significant effect at equimolar concentration (**Figures S1** and **S2**, Supplementary material). The antagonists applied alone did not promote c-Fos accumulation.

**Figure 7:**
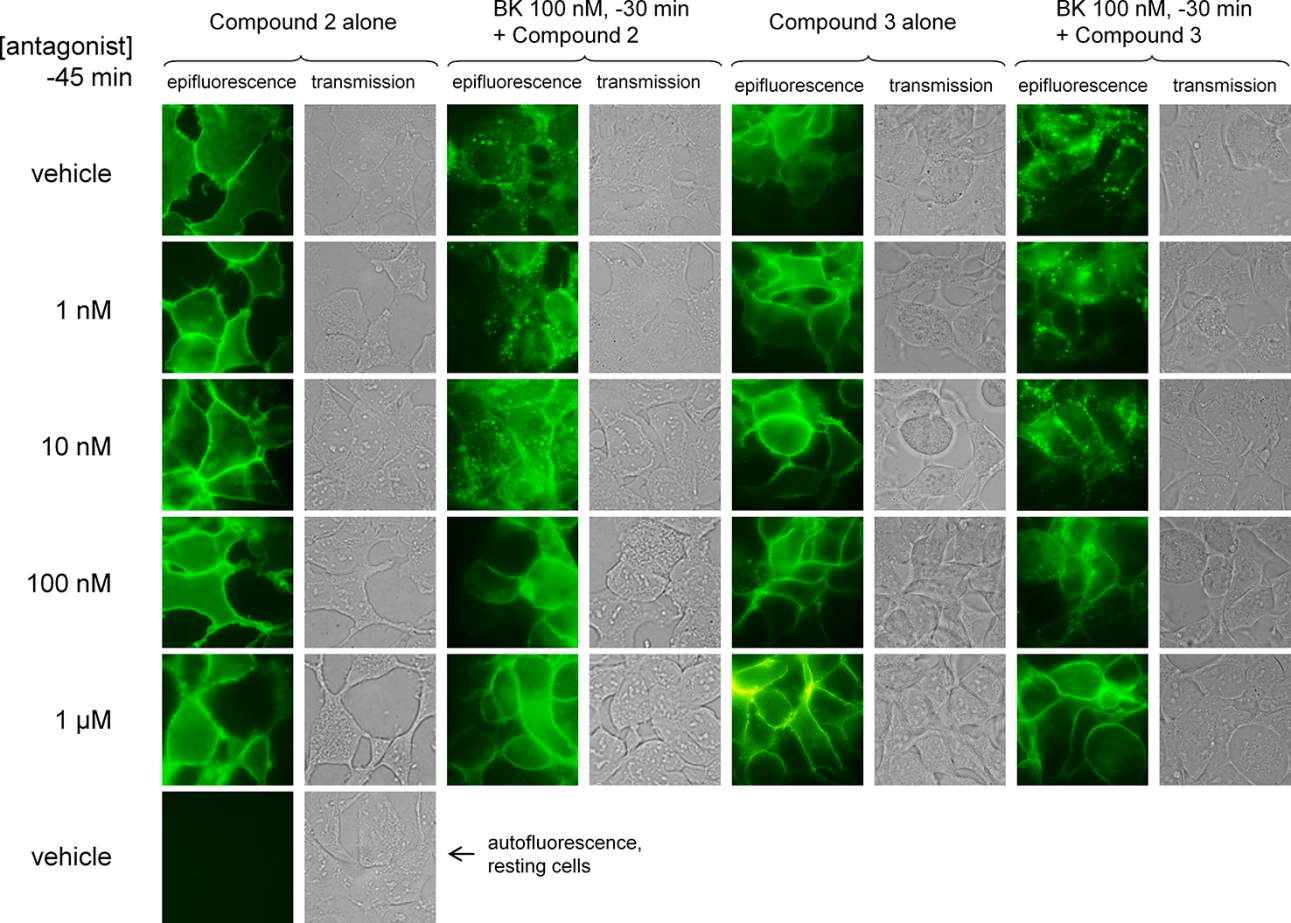
Endocytosis of the fluorescent anti-myc 4A6 monoclonal antibody by intact HEK 293a cells expressing myc-tagged human B_2_ receptors (h myc-B2) treated as indicated at 37°C (BK 0 or 100 nM, the antagonists Compound 2 or Compound 3 at the indicated concentrations 15 min before). The intact cells had been labeled with the anti-myc tag antibodies prior to stimulation (see *Methods*). Each field is shown as a pair of images: epifluorescence in intact cells (left); transmission (right). Original magnification 1,000 ×; the sides of each square field are 120 µm long. Cells incubated without the antibody were photographed using the same settings to evaluate the autofluorescence.

BK and its B_2_ receptor constitute an excellent example of a cycling system of ligand-GPCR complex, starting with agonist-induced receptor phosphorylation by GPCR kinases (Blaukat et al., 2001), receptor mediated ligand endocytosis (Munoz and Leeb-Lundberg, 1992; Charest-Morin et al., 2013), and subsequently, essentially complete receptor recycling at the cell surface upon agonist washout (Blaukat et al., 1996; Bawolak et al., 2007). The anti-myc antibodies, when bound to the myc-B_2_ receptor construction in intact cells, do not functionally antagonize BK but are submitted to agonist-induced endocytosis along with the receptor (Bawolak et al., 2011). This was replicated using a fluorophore-conjugated anti-myc monoclonal antibody, which essentially located at the plasma membrane, but completely internalized in response to BK (100 nM, 30 min) in HEK 293a stably expressing myc-B_2_ receptor (human sequence; **Figure 7**). Despite certain subjectivity in assessing drug effects in this assay, it was observed that pretreating the cells with Compound 2 or Compound 3 (between 1 nM and 1 μM, administered 15 min before BK) inhibited the endocytosis induced by BK (100 nM) in a concentration-dependent manner, with clear efficacy at and above 100 nM for both compounds (**Figure 7**). This represents an agonist/antagonist ratio comparable to one that produces a full antagonism of BK-induced Ca^2+^ mobilisation or venous contraction. The antagonists did not promote significant receptor endocytosis if applied alone.

### Off-Target Profile

Compound 3 was screened at 10 μM against a selected panel of G protein-coupled receptors (GPCRs), ion channels, enzymes and transporters (see **Table S2**, Supplementary material). Compound 3 did not significantly interact (<50% inhibition of binding or functional activity at 10 μM) with most targets (including the bradykinin B_1_ and the angiotensin AT1 receptor), except for the vasopressin receptor 1a (V_1a_ receptor), the corticotropin-releasing factor receptor 1 (CRF_1_ receptor), the prostanoid EP_1_ receptor, the TXA_2_/PGH_2_ receptor (TP receptor), and the neurokinin 1 receptor (NK_1_ receptor). Full concentration–response curves were established for these targets (except for the prostanoid EP1 receptor) in agonist and antagonist mode, and for NK_3_ receptors in addition. Compound 3 was found to show no agonism at these targets, but to be a weak antagonist with K_b_ values in the micromolar range (**Table 8**). These data indicate that Compound 3 is a very selective B_2_ receptor antagonist: at least thousand-fold more potent at the human B_2_ receptor (K_b_ 0.24 nM) as compared to a wide set of off-targets.

**Table 8:** *In vitro* target profile of Compound 3.

	Radioligand competition binding	Functional antagonism		
**GPCR**	**% Inhibition at 10 µM**	**IC_50_ (µM)**	**K_b_ (µM)**	Fold selectivity over the human B_2_ receptor*
V1a	84	9.1	0.31	1,292
CRF1	74	10	3.6	15,000
TP	62	3.6	0.51	2,125
NK1	83	17	2.4	10,000
NK2	21	–	–	–
NK3	6.1	17	1.6	6,667
EP1	80	–	–	–

*Relative to the K_b_ at the human B_2_ receptor (0.24 nM).

### Caco-2 Permeability Assay

We measured the permeability of Compound 1, Compound 2, and Compound 3 across the polarized human colon carcinoma Caco-2 cell monolayer. This bidirectional assay is a standard test that allows a first prediction of absorption rate across human intestinal epithelial cells. Compound 1, Compound 2 and Compound 3 displayed an apparent permeation rate of 5.5 x 10^-6^ cm/s, 3.9 x 10^-6^ cm/s and 17.3 x 10^-6^ cm/s in apical to basolateral direction and a permeation rate of 20.9 x 10^-6^ cm/s, 29.9 x 10^-6^ cm/s, 17.6 x 10^-6^ cm/s in basolateral to apical direction. With a permeability rate >10^-5^ cm/s, and an efflux ratio of 1.0, Compound 3 is predicted to be highly permeable, and not a substrate of efflux pumps such as P-glycoprotein (P-gp) in contrast to Compound 1 and Compound 2 that showed an efflux ratio of 5.5 and 7.7.

### Microsomal Stability

Compound 1, Compound 2, and Compound 3 have been evaluated in a rat liver microsome assay fortified with the phase I metabolism cofactor NADPH. Verapamil was used as the positive control and showed a degradation in the range of 97.00 to 99.97% within 30 min. With a CL_int_ of 960 μL/min/mg protein and 497 μL/min/mg protein Compound 1 as well as Compound 2 revealed a very low stability in the rat microsome assay. Compound 3 showed with a CL_int_ of 27.9 μL/min/mg protein a substantially lower conversion rate and thus potentially a largely improved metabolic stability.

## Discussion

This paper describes the characterization of the highly potent B_2_ receptor antagonist Compound 3 as a representative of a novel chemical class. The difluoromethoxy, the 1-methyl-1*H*-1,2,4-triazol-5-yl, the *m*-chlorofluorophenyl as well as the *S*-configurated benzylic methyl group of compound 3 were identified as key structural elements for achieving high permeability, metabolic stability and activity towards the B_2_ receptor (**Figure 1**). In the patent literature, only six B_2_ receptor antagonists have been described previously that feature a 1-methyl-1*H*-1,2,4-triazol-5-yl group (Gibson et al., 2008; Gibson et al., 2010) with Compound 1 being the closest analog to Compound 3. In comparison to the prior art Compound 1, Compound 3 showed a 5.2-fold improved potency on the B_2_ receptor in the calcium mobilization assay and a 34-fold improved intrinsic clearance in the rat microsomal stability assay. With an efflux ratio of 1.0 in the Caco-2 assay Compound 3 is predicted to be not a substrate of efflux pumps such as P-gp whereas with an efflux ratio of 5.5 Compound 1 seems obviously to be a substrate of efflux pumps. In order to demonstrate the contribution of the novel structural elements (*m*-chlorofluorophenyl group in combination with the defined stereochemical configuration of the benzylic stereocenter) of the new B_2_ antagonists series, the comparator Compound 2 has been synthesized. Compound 2 is furnished with the identical structural elements as Compound 3 except for the pyridine core and the undefined stereo center, both of which are distinctive features of the previously described series (Gibson et al., 2008; Gibson et al., 2009; Gibson et al., 2010). With a 4.0-fold lower potency on the B_2_ receptor, a 18-fold lower intrinsic clearance in the rat microsomal stability assay and a 7.7-fold higher efflux ratio in the Caco-2 permeability assay the comparator Compound 2 clearly revealed a substantially inferior profile when compared with Compound 3.

The potencies of the compounds to antagonize BK-induced Ca^2+^ mobilization at recombinant human B_2_ receptors in CHO cells were very similar to the potencies to antagonize BK-induced contraction at endogenous human B_2_ receptor in the umbilical vein model (K_b_ values in nM and pA_2_ value converted into nM, respectively): Compound 1 (1.24 and 2.63 nM), Compound 2 (0.95 and 0.95 nM), Compound 3 (0.24 and 0.21 nM) and icatibant (2.81 and 8.71 nM). This alignment between these 2 very different assays supports the high potency of Compound 3 at the human B_2_ receptor and confirms the superior potency towards the precedent B_2_ antagonists.

Interestingly, in radioligand binding assays using membranes of a mammalian cell line that express recombinant human B_2_ receptors, the prior art Compound 1 and Compound 2 showed about 7-fold lower affinity for the human B_2_ receptor as compared to Compound 3 and icatibant. In contrast to the 10-40-fold higher functional B_2_ receptor antagonist potency of Compound 3 as compared to icatibant, in this assay, icatibant and Compound 3 showed a similar affinity. The latter is not well understood, but may be related to the low ionic strength applied in the binding assay which may (1) produce an artificially high affinity for [^3^H]BK for the human B2 receptor, and (2) increase artificially the affinity of icatibant for this receptor because these conditions increase the protein-peptide interactions, but not the protein-small molecule interaction.

Species selectivity has been a hurdle in the development of B_2_ receptor antagonists (Leeb-Lundberg et al., 2005). In some cases, rodent specific compounds were discovered, not suitable for further therapeutic development (Hess et al., 1994; Jones et al., 1999; Burgess et al., 2000; Marceau et al., 2003). We here describe that Compound 3 shows high antagonist potency at the human and monkey B_2_ receptors but is several hundred fold less active at rodent and dog B_2_ receptors. This finding is not unexpected, considering the relatively low amino acid sequence identity between B_2_ receptors from human and mouse (84%, Hess et al., 1994), rat (82%, Ma et al., 1994), and dog (81%, Hess et al., 2001). Indeed, several amino acids in the B_2_ receptor known to be engaged in the binding of peptides or small molecule ligands differ between human and other species (Leeb-Lundberg et al., 2005).

The small molecule compounds tested herein are completely devoid of agonist activity in all bioassays: Ca^2+^ mobilization, venous contraction and c-Fos activation. This is of relevance since small molecule partial agonists of the B_2_ receptor have been described in the past, notably Fujisawa's compound 47a, a very active compound able to contract the human umbilical vein (Bawolak et al., 2009). Furthermore, agonist activity at the B_2_ receptor could be associated with injection pain, as hypothesized for icatibant (Floccard et al., 2012).

Compound 3 presumably inhibits all types of B_2_ receptor-initiated signaling: in the persistent contraction of the vascular smooth muscle, protein kinase C (PKC) is involved, as well as rho kinase and myosin light chain kinase and phosphatase (Liu and Khalil, 2018); PKC, the MEK1 and ERK1/2 MAP kinases are involved in c-Fos induction (Bawolak et al., 2009) and the agonist-induced B_2_ receptor endocytosis is dependent on GRKs and β-arrestins (Charest-Morin et al., 2013).

Compound 1, Compound 2, and Compound 3 are competitive B_2_ receptor antagonists, as indicated by the surmountable behavior in the human umbilical vein assay. Indeed, very high concentrations of BK can fully overcome the antagonism of the small molecules, showing no reduction of BK E_max_ value in the presence of antagonist. Despite this undisputable competitive signal, the Schild plot slopes of the small molecule antagonist were somewhat inferior to unity (**Table 4**), possibly indicating a distortion caused by a lack of equilibrium at the B_2_ receptor level due to intracellular entry of the drugs at higher concentrations. Cell permeability can be expected of small molecules, this in contrast to the heavier and hydrophilic peptide icatibant.

Our reversibility experiments in the human umbilical vein contraction model (**Figure 6**), indicate that following Compound 3 treatment and washout, the BK potency was reduced when pretreatment was performed at a concentration of 100 nM, but not at 10 and 1 nM Compound 3. Clearly, Compound 3 bound in a reversible fashion to the B_2_ receptor, as is obvious by the full efficacy of high concentrations of BK (fully surmountable behavior seen in the 3 h concentration response curve). Therefore we hypothesize that cell permeability of Compound 3 may also here be a plausible explanation of the slower/partial reversibility seen at high concentration of this antagonist (100 nM, **Figure 6A**). Perhaps the compound enters the intracellular space, and is released slowly after washing. Similar partial/slow reversibility was described for the small molecule B_2_ receptor antagonist MEN16132 (Meini et al., 2009).

Compound 2 and Compound 3 inhibited endocytosis of the human B_2_ receptor expressed in HEK 293a cells, in a concentration-dependent manner, with clear efficacy at and above 100 nM for both compounds (**Figure 7**). The block of BK-induced internalization is a direct consequence of their competitive interaction with BK. BK-induced internalization of the B_2_ receptor population in cells is fully reversible and not associated with receptor degradation (Blaukat et al., 1996; Bawolak et al., 2007). Further, there is evidence that the naturally expressed B_2_ receptor is long-lived at the cell surface, relative to the rapid clearance of the highly regulated B_1_ receptors (Fortin et al., 2003; Enquist et al., 2007). By inhibiting B_2_ receptor endocytosis, B_2_ receptor antagonists may be expected to increase surface levels of the B_2_ receptor, and may theoretically be expected to elicit a BK-mediated rebound effect following washout or metabolism of the small molecules. However, there is currently no evidence of antagonist-induced upregulation of B_2_ receptor expression, likely indicating that the synthesis rate of the B_2_ receptor is slow (Marceau et al., 1999; Khosravani et al., 2015), and no indication of an increased BK potency after treatment with a B_2_ receptor antagonist and washout (Gobeil et al., 1996 and present **Figure 6**). In man, it was shown that the B_2_ receptor antagonist icatibant retains its efficacy in patients with hereditary angioedema, even after 5 consecutive doses (Baş et al., 2013; Malbrán et al., 2014). Together these data point to the maintenance of antagonist activity following long-term treatment.

Compound 3, was further characterized for its off-target profile. We found that out of a list of 138 molecular targets, including GPCRs, receptors, ion channels, enzymes and transporters, Compound 3 only interacted with a handful of GPCRs, and only at micromolar concentrations. Compound 3 has no affinity for the human B_1_ receptor. Comparing K_b_ values across targets, Compound 3 is shown to be a highly selective B_2_ receptor antagonist with over 1,000 - fold selectivity versus the following human receptors CRF1, NK1, NK3, thromboxane TP, prostanoid EP1 and vasopressin V1a (i.e. a hitrate of 4.3%). Interestingly, although Compound 3 is an antagonist of U-44069-induced Ca^2+^ mobilization at recombinant human TP receptors (IC_50_ value 3.6 μM), when tested against U-46619-induced contractile response mediated by the endogenous human prostanoid TP receptors in the umbilical vein model, no functional antagonism was observed up to 1 μM, confirming the low potency. Also of relevance is the low antagonist potency of Compound 3 for the neurokinin receptors NK1 and NK3 (IC_50_ values were twice 17 μM), and the low affinity for NK2 (21% inhibition of radioligand binding at 10 μM) this is striking as icatibant binds potently to NK2 receptors with an IC_50_ value of 420 nM (FDA Pharmacology Review Icatibant).

The discovery of Compound 3, with picomolar antagonist potency and high specificity for the human B_2_ receptor and predicted oral bioavailability, has provided a significant stepping stone towards the development of a widely usable potent and selective, orally bioavailable B_2_ receptor antagonist. PHA-022121, currently in phase 1 clinical development, is a close analogue of Compound 3.

## Data Availability Statement

The datasets generated for this study are available on request to the corresponding author.

## Ethics Statement

The studies involving human participants were reviewed and approved by Comité d'éthique de la recherche, CHU de Québec-Université Laval. The patients/participants provided their written informed consent to participate in this study.

## Author Contributions

FM designed experiments involving the umbilical vein contractility assay, analyzed results and drafted the corresponding parts of the manuscript. XC-M executed experiments related to signaling in HEK 293a cells and performed preliminary analyses. WK executed and analyzed Calcium mobilization assays with HEK293 cells. AL designed the *in vitro* signal transduction experiments for recombinant B_2_ receptors from different species, the off-target screening panel, analyzed results and wrote related sections to the manuscript. BL collected and summarized data and contributed to the manuscript writing. CG, H-DA and JS designed Compound 3, supervised the synthesis of Compound 1, Compound 2, and Compound 3 and drafted the corresponding parts of the manuscript. All authors contributed to the article and approved the submitted version.

## Funding

The authors declare that this study received funding from Pharvaris Netherlands B.V. XC-M held a Studentship award from *Fonds de recherche Santé du Québec*.

## Conflict of Interest

AL, BL, and JK are consultants for Pharvaris Netherlands B.V. FM served as a consultant and received research funds from Pharvaris Netherlands B.V. CG, H-DA, JS, and WK were employed by AnalytiCon Discovery GmbH.

The remaining author declares that the research was conducted in the absence of any commercialor financial relationships that could be construed as a potential conflict of interest.

The authors declare that this study received funding from Pharvaris Netherlands B.V. The funder was involved in the decision to submit the paper for publication. The funder had no role in the study design, collection, analysis, interpretation of data, or the writing of this article.
