# Supplementary material for: In Vitro Pharmacological Profile of a New Small Molecule Bradykinin B2 Receptor Antagonist
Source: Front Pharmacol. 2020 Jun 19;11:916. doi: 10.3389/fphar.2020.00916 (PMC7316994; doi:10.3389/fphar.2020.00916)
Supplement: Supplementary file 1 [file DataSheet_1.pdf]

Supplementary Material

In vitro pharmacological profile of a new small molecule bradykinin B_2 receptor antagonist

Anne Lesage, Christoph Gibson, François Marceau, Horst-Dieter Ambrosi, Jörn Saupe, Werner Katzer, Brigitte Loenders, Xavier Charest-Morin, Jochen Knolle.

*** Correspondence:** A. Lesage: anne.lesage@pharvaris.com

Supplementary Methods: Immunoblots for c-Fos signaling

The construction of myc-tagged B_2 receptor expression vector containing the human receptor sequence has been recently reported, as well as the derivation of HEK293a cell lines that stably express the construction (Charest-Morin et al., 2015). Immunoblotting experiments, conducted as described (Koumbadinga et al., 2010), were used to study the effect of selected antagonists on BK-induced signaling. Briefly, HEK 293a cells stably expressing the human myc-B_2 receptor were stimulated with BK agonists for 60 min at 37°C, with or without a pretreatment with the antagonist (applied 15 min before BK). Total cell extracts were immunoblotted to detect c-Fos expression using the K-25 rabbit polyclonal antibodies (Santa Cruz Biotechnology, Dallas, TX; dilution 1:50,000). In these experiments, β-actin was used as a loading control and was detected using the monoclonal anti-β-actin antibody, clone AC-15 (Sigma-Aldrich; dilution 1:50,000).

Supplementary References

Charest-Morin X, Raghavan A, Charles ML, Kolodka T, Bouthillier J, Jean M, et al. Pharmacological effects of recombinant human tissue kallikrein on bradykinin B2 receptors. *Pharmacol Res Perspect* (2015) 3:e00119. doi: 10.1002/prp2.119

Koumbadinga GA, Bawolak MT, Marceau E, Adam A, Gera L, Marceau F. A ligand-based approach to investigate the expression and function of angiotensin converting enzyme in intact human umbilical vein endothelial cells. *Peptides* (2010) 31,1546-54. doi: 10.1016/j.peptides.2010.04.027

Figure S1. Signaling response to BK, as measured by c-Fos accumulation, in HEK 293a cells stably expressing myc-tagged human B_2 receptors (h myc-B2) or in non-transfected (nt) cells and effect of the antagonists Compound 2 (A) or Compound 3 (B) given alone or in combination with BK (the agonist BK was applied 60 min before extraction and the antagonist, 75 min before extraction). Replicate number represented by *n*. In panel A, c-Fos densitometry values significantly differed between them (ANOVA $P < 0.01$). Pairs of values were compared using Tukey's multiple comparison test: * $P < 0.05$ *vs.* control of receptor-expressing cells; † $P < 0.05$ and †† $P < 0.01$ *vs.* the effect of BK 10 nM in receptor-expressing cells. In panel B, c-Fos densitometry values significantly differed between them (ANOVA, $P < 0.01$). Pairs of values were compared using Tukey's multiple comparison test: ** $P < 0.01$ *vs.* control of receptor-expressing cells; †† $P < 0.01$ *vs.* the effect of BK 10 nM in receptor-expressing cells. Values are the means ± S.E.M. of the number of determinations indicated by *n*. The individual blots are shown in Figure S2.

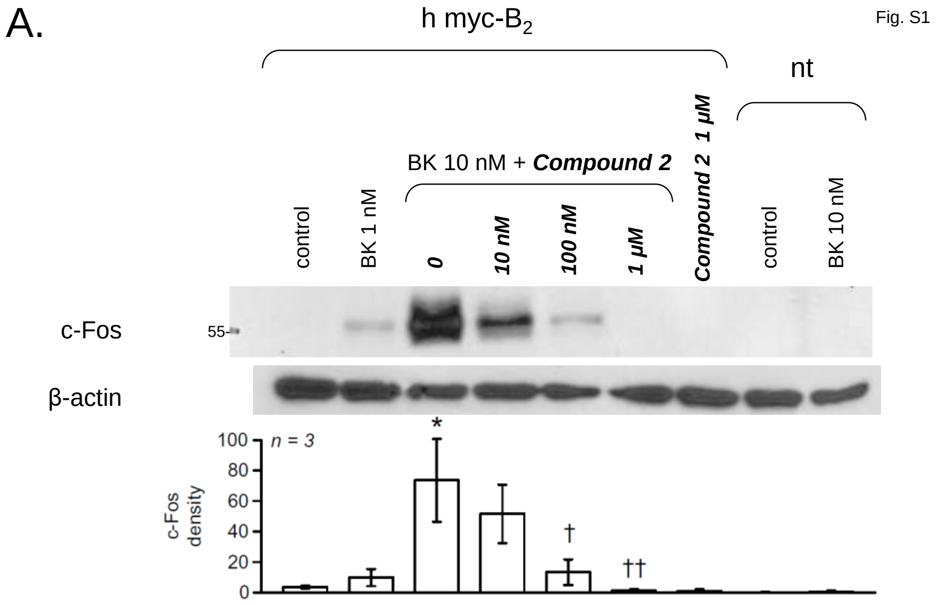

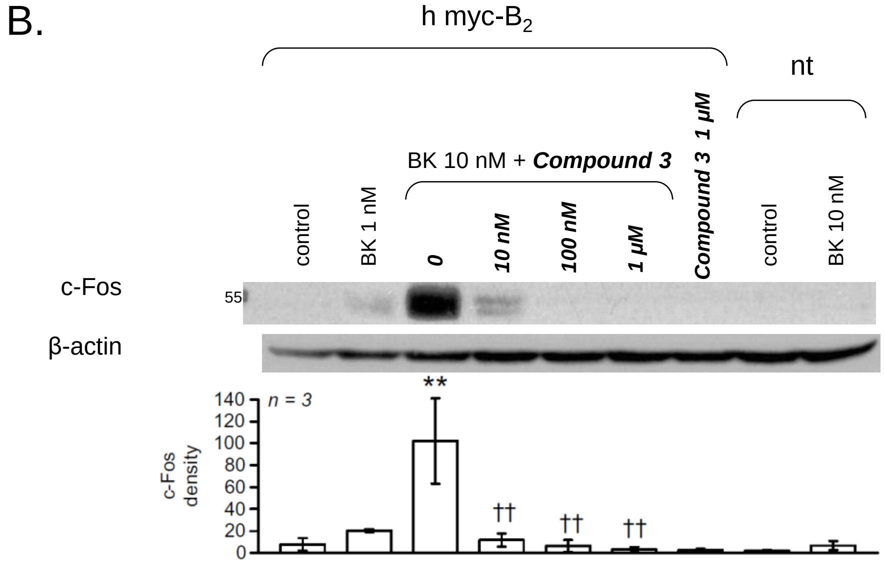

Figure S2. Full scans of the entire original gels of western blot images represented in Figure S1. Lanes: same order as in Figure S1. Rows: 3 independent experiments per panel A and B.

Fig. S2

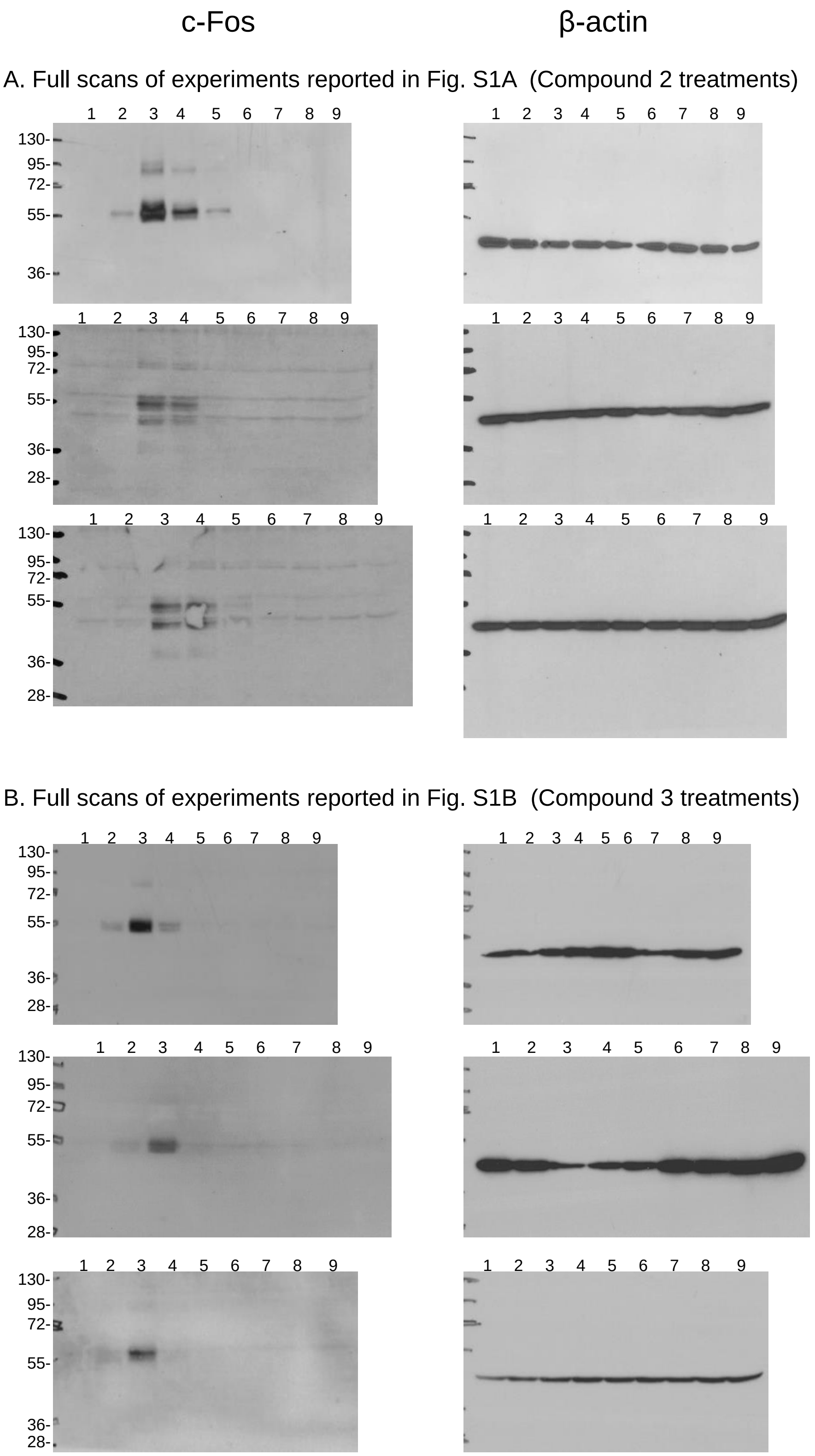

Table S1. Maximal BK-induced response in absolute force units (grams-weight) ± S.E.M. for each set of tissues and test item concentration used to assess antagonism. The number of determinations is the same as in the pooled dose-response curves (Fig. 3). ANOVA followed by Dunnett's test applied to lines of data (comparison with the common vehicle controls) did not show significant differences.

Concentration → Compound ↓	Vehicle	1 nM	10 nM	100 nM	1 µM	10 µM
Icatibant	1.90 ± 0.25	---	1.47 ± 0.19	1.50 ± 0.27	1.40 ± 0.22	1.27 ± 0.17
Compound 1	1.15 ± 0.23	---	1.48 ± 0.24	2.08 ± 0.28	1.46 ± 0.20	1.67 ± 0.28
Compound 2	2.25 ± 0.40	---	2.07 ± 0.30	2.26 ± 0.42	2.00 ± 0.32	1.82 ± 0.18
Compound 3	1.52 ± 0.20	1.75 ± 0.32	2.29 ± 0.24	2.42 ± 0.36	1.25 ± 0.14	---

Table S2. Profiling of Compound 3 across a panel of molecular targets

1. Conditions for radioligand binding assays conducted with Compound 3

**Target**	**Source**	**Assay conditions**	**Labelled ligand**<br>**Name**<br>**concentration**<br>**K_d value**	**Non-specific ligand**<br>**Name**<br>**concentration**	**Compound 3 % Inhibition @ 10 µM**
Adenosine A_1	Human recombinant CHO cells	60 minutes @ RT	[^{3}H] DPCPX<br>1 nM<br>1.7 nM	DPCPX<br>1 µM	12.4
Adenosine A_{2A}	Human recombinant HEK-293 cells	120 minutes @ RT	[^{3}H] CGS 21680<br>6 nM<br>27 nM	NECA<br>10 µM	9.8
Adenosine A_{2B}	Human recombinant HEK-293 cells	60 minutes @ RT	[^{3}H] CPX<br>5 nM<br>65 nM	NECA<br>100 µM	3.6
Adenosine A_3	Human recombinant HEK-293 cells	120 minutes @ RT	[^{125}I] AB-MECA<br>0.15 nM<br>0.22 nM	IB-MECA<br>1 µM	39.8
Adrenergic α_{1A}	Human recombinant CHO cells	60 minutes @ RT	[^{3}H] Prazosin<br>0.1 nM<br>0.1 nM	Epinephrine<br>0.1 mM	13.3

**Target**	**Source**	**Assay conditions**	**Labelled ligand**<br>**Name**<br>**concentration**<br>**K_d value**	**Non-specific ligand**<br>**Name**<br>**concentration**	**Compound 3 % Inhibition @ 10 µM**
Adrenergic α_{2A}	Human recombinant CHO cells	60 minutes @ RT	[^{3}H] RX 821002<br>1 nM<br>0.8 nM	(-) Epinephrine<br>100 µM	-1.7
Adrenergic β_1	Human recombinant HEK-293 cells	60 minutes @ RT	[^{3}H] (-)CGP 12177<br>0.3 nM<br>0.39 nM	Alprenolol<br>50 µM	0.3
Adrenergic β_2	Human recombinant CHO cells	120 minutes @ RT	[^{3}H] (-)CGP 12177<br>0.3 nM<br>0.15 nM	Alprenolol<br>50 µM	3.0
Adrenergic β_3	Human recombinant HEK-293 cells	90 minutes @ 25°C	[^{125}I] Cyanopindolol<br>0.5 nM<br>1.5 nM	Alprenolol<br>1000.0 µM	8.1
Androgen AR	LNCaP cells (cytosol)	24 hours @ 4°C	[^{3}H] Methyltrienolone<br>1 nM<br>0.8 nM	Testosterone<br>1 µM	0.4
Angiotensine AT_1	Human recombinant HEK-293 cells	120 minutes @ 37°C	[^{125}I] [Sar^1, Ile^8]-ATII<br>0.05 nM<br>0.05 nM	Angiotensin-II<br>10 µM	-4.1
Angiotensine AT_2	Human recombinant HEK-293 cells	4 hours @ 37°C	[^{125}I] CGP 42112A<br>0.01 nM<br>0.01 nM	Angiotensin-II<br>1 µM	3.5

**Target**	**Source**	**Assay conditions**	**Labelled ligand**	**Non-specific ligand**	**Compound 3 % Inhibition @ 10 µM**
			**Name concentration K_d value**	**Name concentration**	
Apelin APJ	Human recombinant CHO cells	120 minutes @ RT	[^{125}I] [Glp^{65}, Nle^{75}, Tyr^{77}]-apelin-13<br>0.03 nM<br>0.06 nM	Apelin-13<br>1 µM	-0.4
Benzodiazepine BZD (central)	Rat cerebral cortex	60 minutes @ 4°C	[^{3}H] Flunitrazepam<br>0.4 nM<br>2.1 nM	Diazepam<br>3 µM	-25.8
Benzodiazepine BZD (peripheral)	Rat heart	15 minutes @ RT	[^{3}H] PK 11195<br>0.2 nM<br>1.8 nM	PK 11195<br>10 µM	42.3
Bombesin BB1	Human recombinant CHO cells	60 minutes @ RT	[^{125}I] [Tyr^{4}]-bombesin<br>0.1 nM<br>0.1 nM	Neuromedin B<br>1 µM	6.7
Bombesin BB2	Human recombinant HEK-293 cells	60 minutes @ RT	[^{125}I] [Tyr^{4}]-bombesin<br>0.04 nM<br>0.04 nM	Bombesin<br>1 µM	-1.4
Bradykinin B1	Human recombinant CHO cells	60 minutes @ RT	[^{3}H] desArg^{10}-KD<br>0.35 nM<br>0.085 nM	desArg^{9}[Leu^{8}]-BK<br>10 µM	0.5
Calcitonin gene related peptide CGRP	Human recombinant CHO cells	90 minutes @ RT	[^{125}I] hCGRPα<br>0.03 nM<br>0.06 nM	hCGRPα<br>1 µM	-26.1

Target	Source	Assay conditions	Labelled ligand<br>Name<br>concentration<br>K_d value	Non-specific ligand<br>Name<br>concentration	Compound 3<br>% Inhibition<br>@ 10 µM
Calcium Ca^{2+} channel (L-dihydropyridine site)	Rat cerebral cortex	90 minutes @ RT	[^{3}H] Nitrendepine<br>0.1 nM<br>0.18 nM	Nitrendipine<br>1 µM	7.3
Cannabinoid CB_1	Human recombinant CHO cells	120 minutes @ 37°C	[^{3}H] CP 55940<br>0.5 nM<br>3.5 nM	WIN 55212-2<br>10 µM	-3.0
Cannabinoid CB_2	Human recombinant CHO cells	120 minutes @ 37°C	[^{3}H] WIN 55212-2<br>0.8 nM<br>1.5 nM	WIN 55212-2<br>5 µM	-17.7
Chemokine CCR1	Human recombinant HEK-293 cells	120 minutes @ RT	[^{125}I] MIP-1α<br>0.01 nM<br>0.02 nM	MIP-1α<br>100 nM	-9.6
Chemokine CCR2B	Human recombinant CHO-K1 cells	pH 7.4<br>60 minutes @ 25°C	[^{125}I] MCP-1<br>0.10 nM<br>0.063 nM	MCP-1<br>0.030 µM	1
Chemokine CXCR2 (IL-$8R_B$)	Human recombinant CHO-K1 cells	pH 7.4<br>60 minutes @ 25°C	[^{125}I] IL-8<br>15.0 pM<br>12.0 pM	IL-8<br>10 nM	17
Chemokine CXCR4	Human recombinant Chem-1 cells	pH 7.4<br>90 minutes @ 25°C	[^{125}I] SDF-1α<br>0.030 nM<br>0.096 nM	SDF-1α<br>0.030 µM	10

**Target**	**Source**	**Assay conditions**	**Labelled ligand**	**Non-specific ligand**	**Compound 3 % Inhibition @ 10 µM**
			**Name concentration K_d value**	**Name concentration**	
Chloride Cl^- channel (GABA-gated)	Rat cerebral cortex	120 minutes @ RT	[^{35}S] TBPS<br>3 nM<br>14.6 nM	Picrotoxin<br>20 µM	32.9
Cholecystokinin CCK_1	Human recombinant CHO cells	60 minutes @ RT	[^{125}I] CCK-8s<br>0.08 nM<br>0.24 nM	CCK-8s<br>1 µM	2.7
Cholecystokinin CCK_2 (CCK_B)	Human recombinant Chem-1 cells	pH 7.4<br>3 hours @ 25°C	[^{125}I] CCK-8<br>0.050 nM<br>0.069 nM	Sincalide<br>1.0 µM	1
Corticotropin-releasing hormone receptor CRF1	Human recombinant CHO cells	120 minutes @ RT	[^{125}I] Sauvagine<br>0.075 nM<br>0.12 nM	Sauvagine<br>0.5 µM	73.5
Cysteinyl leukotriene receptor $CysLT_1$	Human recombinant CHO cells	60 minutes @ RT	[^{3}H] LTD_4<br>0.3 nM<br>0.24 nM	LTD_4<br>1 µM	-2.2
Cysteinyl leukotriene receptor $CysLT_2$	Human recombinant HEK-293 cells	60 minutes @ RT	[^{3}H] LTD_4<br>1 nM<br>1.5 nM	LTD_4<br>1 µM	-6.0
Dopamine D_1	Human recombinant CHO cells	60 minutes @ RT	[^{3}H] SCH 23390<br>0.3 nM<br>0.2 nM	SCH 23390<br>1 µM	6.9

Target	Source	Assay conditions	Labelled ligand	Non-specific ligand	Compound 3 % Inhibition @ 10 µM
			Name<br>concentration<br>K_d value	Name<br>concentration	
Dopamine D_{2S}	Human recombinant<br>HEK-293 cells	60 minutes @ RT	[^{3}H] 7-OH-DPAT<br>1 nM<br>0.68 nM	Butaclamol<br>10 µM	-2.7
Dopamine D_3	Human recombinant<br>CHO cells	60 minutes @ RT	[^{3}H] Methylspiperone<br>0.3 nM<br>0.085 nM	(+) butaclamol<br>10 µM	4.8
Dopamine $D_{4.2}$	Human recombinant<br>CHO-K1 cells	pH 7.4<br>2 hours @ 25°C	[^{3}H] Spiperone<br>0.50 nM<br>0.32 nM	Haloperidol<br>10.0 µM	2
Dopamine transporter	Human recombinant<br>CHO cells	120 minutes @ 4°C	[^{3}H] BTCP<br>4 nM<br>4.5 nM	BTCP<br>10 µM	2.2
Endothelin ET_A	Human recombinant<br>CHO cells	120 minutes @ 37°C	[^{125}I] Endothelin-1<br>0.03 nM<br>0.03 nM	Endothelin-1<br>100 nM	-2.1
Endothelin ET_B	Human recombinant<br>CHO cells	120 minutes @ 37°C	[^{125}I] Endothelin-1<br>0.03 nM<br>0.04 nM	Endothelin-1<br>0.1 µM	7.2
Estrogen ERα	Human recombinant sf9 cells	120 minutes @ RT	[^{3}H] Estradiol<br>0.5 nM<br>0.20 nM	Diethylstilbestrol<br>1 µM	-19.5

Target	Source	Assay conditions	Labelled ligand	Non-specific ligand	Compound 3 % Inhibition @ 10 µM
			**Name concentration K_d value**	**Name concentration**	
Galanin GAL_1	Human recombinant HEK-293 cells	60 minutes @ RT	[^{125}I] Galanin<br>0.1 nM<br>0.1 nM	Galanin<br>1 µM	22.4
Galanin GAL_2	Human recombinant CHO cells	120 minutes @ RT	[^{125}I] Galanin<br>0.05 nM<br>0.63 nM	Galanin<br>1 µM	6.2
Gamma-aminobutyric acid $GABA_A$, muscimol, central	Wistar rat brain (minus cerebellum)	pH 7.4<br>10 minutes @ 4°C	[^{3}H] Muscimol<br>1.0 nM<br>3.80 nM	Muscimol<br>0.10 µM	12
Gamma-aminobutyric acid $GABA_{B1A}$	Human recombinant CHO-K1 cells	pH 7.4<br>3 hours @ 25°C	[^{3}H] CGP-54626<br>4.0 nM<br>3.30 nM	GABA<br>3.0 mM	8
Gamma-aminobutyric acid $GABA_{B1B}$	Human recombinant CHO-K1 cells	pH 7.4<br>3 hours @ 25°C	[^{3}H] CGP-54626<br>4.0 nM<br>3.30 nM	GABA<br>3.0 mM	9
Glucagon	Human recombinant CHO cells	120 minutes @ RT	[^{125}I] Glucagon<br>0.025 nM<br>0.069 nM	Glucagon<br>1 µM	-0.6
Glucocorticoid GR	IM-9 cells (cytosol)	6 hours @ 4°C	[^{3}H] Dexamethasone<br>1.5 nM<br>1.5 nM	Triamcinolone<br>10 µM	-4.1

Target	Source	Assay conditions	Labelled ligand<br>Name<br>concentration<br>K_d value	Non-specific ligand<br>Name<br>concentration	Compound 3<br>% Inhibition<br>@ 10 µM
Glutamate ionotropic, AMPA	Rat cerebral cortex	60 minutes @ 4°C	[^{3}H] AMPA<br>8 nM<br>82 nM	L-glutamate<br>1 mM	3.0
Glutamate ionotropic, NMDA	Rat cerebral cortex	60 minutes @ 4°C	[^{3}H] CGP 39653<br>5 nM<br>23 nM	L-glutamate<br>100 µM	5.1
Glutamate, metabotropic, $mGlu_5$	Human recombinant CHO-K1 cells	pH 7.4<br>2 hours @ 25°C	[^{3}H] Quisqualic acid<br>0.030 µM<br>0.026 µM	L-glutamic acid<br>1.0 mM	-8
Glutamate, NMDA, phencyclidine	Wistar rat cerebral cortex	pH 7.4<br>45 minutes @ 25°C	[^{3}H] TCP<br>4.0 nM<br>8.40 nM	Dizocilpine ((+)-MK-801)<br>1.0 µM	-2
Glycine, strychnine-sensitive	Wistar rat spinal cord	pH 7.1<br>10 minutes @ 4°C	[^{3}H] Strychnine<br>10 nM<br>13.0 nM	Glycine<br>1.0 mM	-1
Gonadotropin-releasing hormone	Human recombinant Chem-1 cells	pH 7.4<br>60 minutes @ 25°C	[^{125}I] [D-Trp^6]-LH-RH<br>0.050 nM<br>0.31 nM	[D-Trp^6]-LH-RH<br>1.0 µM	5
Growth hormone secretagogue (GHS, ghrelin)	Human recombinant CHO-K1 cells	pH 7.4<br>60 minutes @ 25°C	[^{125}I] Ghrelin (human)<br>0.030 nM<br>0.038 nM	Ghrelin (human)<br>0.10 µM	6

**Target**	**Source**	**Assay conditions**	**Labelled ligand**	**Non-specific ligand**	**Compound 3 % Inhibition @ 10 µM**
			**Name concentration K_d value**	**Name concentration**	
Histamine H_1	Human recombinant HEK-293 cells	60 minutes @ RT	[^{3}H] Pyrilamine<br>1 nM<br>1.7 nM	Pyrilamine<br>1 µM	2.5
Histamine H_2	Human recombinant CHO cells	120 minutes @ RT	[^{125}I] APT<br>0.075 nM<br>2.9 nM	Tiotidine<br>100 µM	-26.5
Histamine H_3	Human recombinant CHO cells	60 minutes @ RT	[^{3}H] N^α-Me-histamine<br>1 nM<br>0.32 nM	(R)α-Me-histamine<br>1 µM	6.2
Histamine H_4	Human recombinant HEK-293 cells	60 minutes @ RT	[^{3}H] Histamine<br>10 nM<br>7.6 nM	Imetit<br>1 µM	-2.8
Kainate	Rat cerebral cortex	60 minutes @ 4°C	[^{3}H] Kainic acid<br>5 nM<br>19 nM	L-glutamate<br>1 mM	-4.4
Leukotriene B4 receptor BLT_1	Human recombinant CHO cells	60 minutes @ RT	[^{3}H] LTB_4<br>0.2 nM<br>0.2 nM	LTB_4<br>0.2 µM	16.7
Melanin-concentrating hormone receptor MCH_1	Human recombinant CHO cells	60 minutes @ RT	[^{125}I] [Phe^{13}, Tyr^{19}]-MCH<br>0.1 nM<br>1 nM	Human MCH<br>0.1 µM	13.4

Target	Source	Assay conditions	Labelled ligand	Non-specific ligand	Compound 3 % Inhibition @ 10 µM
			Name<br>concentration<br>K_d value	Name<br>concentration	
Melanocortin MC_3	Human recombinant CHO cells	60 minutes @ 37°C	[^{125}I] NDP-α-MSH<br>0.075 nM<br>0.4 nM	NDP-α-MSH<br>1 µM	-8.2
Melanocortin MC_4	Human recombinant CHO cells	120 minutes @ 37°C	[^{125}I] NDP-α-MSH<br>0.05 nM<br>0.54 nM	NDP-α-MSH<br>1 µM	7.1
Melanocortin MC_5	Human recombinant CHO cells	60 minutes @ 37°C	[^{125}I] NDP-α-MSH<br>0.05 nM<br>0.7 nM	NDP-α-MSH<br>1 µM	-9.6
Melatonin MT_1 (ML_{1A})	Human recombinant CHO cells	60 minutes @ RT	[^{125}I] 2-iodomelatonin<br>0.01 nM<br>0.04 nM	Melatonin<br>1 µM	15.3
Melatonin MT_2 (ML_{1B})	Human recombinant CHO cells	120 minutes @ 37°C	[^{125}I] 2-iodomelatonin<br>0.05 nM<br>0.085 nM	Melatonin<br>1 µM	4.8
Monoamino-oxidase MAO-A	Rat cerebral cortex	60 minutes @ 37°C	[^{3}H] Ro 41-1049<br>10 nM<br>14 nM	Clorgyline<br>1 µM	-1.3
Motilin	Human recombinant CHO cells	120 minutes @ RT	[^{125}I] Motilin<br>0.05 nM<br>0.26 nM	[Nleu^{13}]-motilin<br>1 µM	5.0

**Target**	**Source**	**Assay conditions**	**Labelled ligand**	**Non-specific ligand**	**Compound 3 % Inhibition @ 10 μM**
			**Name concentration K_d value**	**Name concentration**	
Muscarinic, oxotremorine M	Wistar rat cerebral cortex	pH 7.4<br>30 minutes @ 37°C	[^{3}H] Oxotremorine M<br>5.0 nM<br>4.80 nM	Oxotremorine M<br>10.0 μM	18
Muscarinic M_1	Human recombinant CHO cells	60 minutes @ RT	[^{3}H] Pirenzepine<br>2 nM<br>13 nM	Atropine<br>1 μM	3.7
Muscarinic M_2	Human recombinant CHO cells	60 minutes @ RT	[^{3}H] AF-DX 384<br>2 nM<br>4.6 nM	Atropine<br>1 μM	-12.4
Muscarinic M_3	Human recombinant CHO cells	60 minutes @ RT	[^{3}H] 4-DAMP<br>0.2 nM<br>0.5 nM	Atropine<br>1 μM	4.2
Muscarinic M_4	Human recombinant CHO cells	60 minutes @ RT	[^{3}H] 4-DAMP<br>0.2 nM<br>0.32 nM	Atropine<br>1 μM	-1.3
Muscarinic M_5	Human recombinant CHO cells	60 minutes @ RT	[^{3}H] 4-DAMP<br>0.3 nM<br>0.3 nM	Atropine<br>1 μM	5.1
Neurokinin NK_1	U373MG uppsala	30 minutes @ RT	[^{125}I]-Substance P LYS3<br>0.05 nM<br>0.04 nM	[Sar^9, Met(O_2)^{11}]-SP<br>1 μM	82.8

Target	Source	Assay conditions	Labelled ligand<br>Name<br>concentration<br>K_d value	Non-specific ligand<br>Name<br>concentration	Compound 3<br>% Inhibition<br>@ 10 µM
Neurokinin NK_2	Human recombinant<br>CHO cells	60 minutes @ RT	[^{125}I] NKA<br>0.1 nM<br>0.12 nM	[Nleu^{10}]-NKA (4-10)<br>300 nM	21.2
Neurokinin NK_3	Human recombinant<br>CHO cells	120 minutes @ RT	[^{3}H] SR 142801<br>0.4 nM<br>0.47 nM	SB 222200<br>10 µM	6.1
Neuropeptide Y_1	SK-N-MC cells<br>(endogenous)	120 minutes @ 37°C	[^{125}I] Peptide YY<br>0.025 nM<br>0.06 nM	NPY<br>1 µM	-2.6
Neuropeptide Y_2	KAN-TYS cells	60 minutes @ 37°C	[^{125}I] Peptide YY<br>0.015 nM<br>0.01 nM	NPY<br>1 µM	-19.1
Neurotensin NT_1	Human recombinant<br>HEK-293 cells	pH 7.4<br>45 minutes @ 25°C	[^{125}I] Neurotensin<br>20.0 pM<br>0.082 nM	Neurotensin<br>1.0 µM	4
Nicotinic acetylcholine<br>α1, bungarotoxin	Human RD cells	2 hours @ 25°C	[^{125}I] α-Bungarotoxin<br>0.60 nM<br>1.10 nM	α-Bungarotoxin<br>1.0 µM	-1
Nicotinic N neuronal<br>α4β2	Human recombinant SH-SY5Y cells	120 minutes @ 4°C	[^{3}H] Cytisine<br>0.6 nM<br>0.3 nM	Nicotine<br>10 µM	-33.6

Target	Source	Assay conditions	Labelled ligand<br>Name<br>concentration<br>K_d value	Non-specific ligand<br>Name<br>concentration	Compound 3<br>% Inhibition<br>@ 10 µM
Norepinephrine transporter	Human recombinant CHO cells	120 minutes @ 4°C	[^{3}H] Nisoxetine<br>1 nM<br>2.9 nM	Desimipramine<br>1 µM	0.9
Opioid δ (DOP)	Human recombinant CHO cells	120 minutes @ RT	[^{3}H] DADLE<br>0.5 nM<br>0.73 nM	Naltrexone<br>10 µM	10.6
Opioid κ (KOP)	Rat recombinant CHO cells	60 minutes @ RT	[^{3}H] U 69593<br>1 nM<br>2 nM	Naloxone<br>10 µM	15.4
Opioid µ (MOP)	Human recombinant HEK-293 cells	120 minutes @ RT	[^{3}H] DAMGO<br>0.5 nM<br>0.35 nM	Naloxone<br>10 µM	13.6
Orexin OX_1	Human recombinant CHO cells	60 minutes @ RT	[^{125}I] Orexin A<br>0.1 nM<br>10 nM	SB 334867<br>1 µM	7.1
Orexin OX_2	Human recombinant HEK-293 cells	180 minutes @ RT	[^{125}I] Orexin A<br>0.04 nM<br>0.2 nM	Orexin-B<br>1 µM	25.9
Opioid nociceptin NOP (ORL1)	Human recombinant HEK-293 cells	60 minutes @ RT	[^{3}H] Nociceptin<br>0.2 nM<br>0.4 nM	Nociceptin<br>1 µM	14.5

Target	Source	Assay conditions	Labelled ligand	Non-specific ligand	Compound 3 % Inhibition @ 10 µM
			Name<br>concentration<br>K_d value	Name<br>concentration	
Oxytocin	Human recombinant<br>Chem-1 cells	pH 7.4<br>2 hours @ 25°C	[^{3}H] Oxytocin<br>0.80 nM<br>0.39 nM	Oxytocin<br>1.0 µM	5
Platelet-activating factor PAF	Human recombinant<br>CHO cells	60 minutes @ RT	[^{3}H] C_{16}-PAF<br>1.5 nM<br>1.5 nM	WEB 23086<br>10 µM	25
Potassium channel hERG	Human recombinant<br>HEK-293 cells	60 minutes @ RT	[^{3}H] Dofetilide<br>3 nM<br>6.6 nM	Terfenadine<br>25 µM	17.8
Potassium channel K_{ATP}	Rat cerebral cortex	60 minutes @ RT	[^{3}H] Glibenclamide<br>0.1 nM<br>0.05 nM	Glibenclamide<br>1 µM	2.3
Potassium channel voltage gated K_V channel	Rat cerebral cortex	60 minutes @ RT	[^{125}I] α-dendrotoxin<br>0.01 nM<br>0.04 nM	α-dendrotoxin<br>50 nM	2.3
Potassium channel (Ca activated) SK_{Ca} channel	Rat cerebral cortex	60 minutes @ 4°C	[^{125}I] Apamin<br>0.007 nM<br>0.007 nM	Apamin<br>100 nM	6.3
Prostacyclin IP (PGI2)	Human recombinant<br>HEK-293 cells	60 minutes @ RT	[^{3}H] Iloprost<br>6 nM<br>8 nM	Iloprost<br>10 µM	-5.0

Target	Source	Assay conditions	Labelled ligand	Non-specific ligand	Compound 3 % Inhibition @ 10 µM
			Name<br>concentration<br>K_d value	Name<br>concentration	
Prostanoid CRTH2	Human recombinant<br>CHO-K1 cells	pH 7.4<br>2 hours @ 25°C	[^{3}H] Prostaglandin D_2<br>1.0 nM<br>4.10 nM	Prostaglandin D_2<br>1.0 µM	3
Prostanoid EP_1	Human recombinant<br>HEK-293 cells	pH 6.0<br>60 minutes @ 25°C	[^{3}H] Prostaglandin E_2<br>1.0 nM<br>5.20 nM	Prostaglandin E_2<br>10.0 µM	80
Prostanoid EP_2	Human recombinant<br>HEK-293 cells	pH 6.0<br>2 hours @ 25°C	[^{3}H] Prostaglandin E_2<br>4.0 nM<br>3.10 nM	Prostaglandin E_2<br>10.0 µM	12
Prostanoid EP_4	Human recombinant<br>Chem-1 cells	pH 6.0<br>2 hours @ 25°C	[^{3}H] Prostaglandin E_2<br>1.0 nM<br>0.69 nM	Prostaglandin E_2<br>10.0 µM	3
Prostanoid FP	Human recombinant<br>HEK-293 cells	pH 6.0<br>60 minutes @ 25°C	[^{3}H] Prostaglandin $F_2α$<br>1.0 nM<br>2.40 nM	Cloprostenol<br>1.0 µM	19
Purinergic receptor P2X	Rat urinary bladder	120 minutes @ 4°C	[^{3}H] α,β-MeATP<br>3 nM<br>2.6 nM	α,β-MeATP<br>10 µM	6.3
Purinergic receptor P2Y	Rat cerebral cortex	60 minutes @ RT	[^{35}S] dATPαS<br>10 nM<br>10 nM	dATPαS<br>10 µM	-4.3

Target	Source	Assay conditions	Labelled ligand	Non-specific ligand	Compound 3 % Inhibition @ 10 µM
			**Name concentration K_d value**	**Name concentration**	
Serotonin 5-HT_{1A}	Human recombinant HEK-293 cells	60 minutes @ RT	[^{3}H] 8-OH-DPAT<br>0.5 nM<br>0.5 nM	8-OH-DPAT<br>10 µM	-4.0
Serotonin 5-HT_{1B}	Rat cerebral cortex	120 minutes @ 37°C	[^{125}I] CYP (+ 30 µM isoproterenol)<br>0.1 nM<br>0.16 nM	Serotonin<br>10 µM	-2.5
Serotonin 5-HT_{2A}	Human recombinant HEK-293 cells	60 minutes @ RT	[^{125}I] (±)DOI<br>0.1 nM<br>0.3 nM	(±)DOI<br>1 µM	-3.8
Serotonin 5-HT_{2B}	Human recombinant CHO cells	60 minutes @ RT	[^{125}I] (±)DOI<br>0.2 nM<br>0.2 nM	(±)DOI<br>1 µM	-8.9
Serotonin 5-HT_{2C}	Human recombinant HEK-293 cells	120 minutes @ 37°C	[^{3}H] Mesulergine<br>1 nM<br>0.5 nM	RS 102221<br>10 µM	5.0
Serotonin 5-HT_{3}	Human recombinant CHO cells	120 minutes @ RT	[^{3}H] BRL 43694<br>0.5 nM<br>1.15 nM	MDL 72222<br>10 µM	2.1
Serotonin 5-$HT_{4\,e}$	Human recombinant CHO cells	60 minutes @ 37°C	[^{3}H] GR 113808<br>0.3 nM<br>0.15 nM	Serotonin<br>100 µM	-0.9

Target	Source	Assay conditions	Labelled ligand	Non-specific ligand	Compound 3 % Inhibition @ 10 µM
			Name<br>concentration<br>K_d value	Name<br>concentration	
Serotonin 5-HT_6	Human recombinant CHO cells	120 minutes @ 37°C	[^{3}H] LSD<br>2 nM<br>1.8 nM	Serotonin<br>100 µM	-4.5
Serotonin 5-HT_7	Human recombinant CHO cells	120 minutes @ RT	[^{3}H] LSD<br>4 nM<br>1.3 nM	Serotonin<br>10 µM	3.3
Serotonin 5-HT transporter	Human recombinant CHO cells	60 minutes @ RT	[^{3}H] Imipramine<br>2 nM<br>1.7 nM	Imiupramine<br>10 µM	-23.4
Sodium Na^+ channel (site 2)	Rat cerebral cortex	60 minutes @ 37°C	[^{3}H] Batrachotoxinin<br>10 nM<br>91 nM	Veratridine<br>300 µM	-3.4
Somatostatin sst1	Human recombinant CHO-S cells	pH 7.4<br>2 hours @ 25°C	[^{125}I] Tyr^{11}-Somatostatin 14<br>0.10 nM<br>0.48 nM	Somatostatin-14<br>1.0 µM	-2
Somatostatin sst2	Human recombinant CHO-K1 cells	pH 7.4<br>4 hours @ 25°C	[^{125}I] Tyr^{11}-Somatostatin 14<br>0.030 nM<br>0.034 nM	Somatostatin-14<br>1.0 µM	-7
Somatostatin sst5	Human recombinant Chem-1 cells	pH 7.4<br>2 hours @ 25°C	[^{125}I] Tyr^{11}-Somatostatin 14<br>0.10 nM<br>0.13 nM	Somatostatin-14<br>1.0 µM	2

Target	Source	Assay conditions	Labelled ligand	Non-specific ligand	Compound 3 % Inhibition @ 10 µM
			**Name concentration K_d value**	**Name concentration**	
Thyroid hormone	Rat liver	1080 minutes @ 4°C	[^{125}I] Triiodothyronine<br>0.03 nM<br>0.034 nM	Triiodothyronine<br>1.0 µM	6.4
Thyrotropin-releasing hormone TRH_1	Human recombinant CHO cells	120 minutes @ 4°C	[^{3}H] Me-TRH<br>2 nM<br>3.9 nM	TRH<br>10 µM	21.7
Urotensin UT	Human recombinant CHO cells	120 minutes @ RT	[^{125}I] Urotensin-II<br>0.1 nM<br>0.29 nM	Urotensin-II<br>3 µM	13.5
Vasoactive intestinal peptide VPAC1 (VIP1)	Human recombinant CHO cells	60 minutes @ RT	[^{125}I] VIP<br>0.04 nM<br>0.05 nM	VIP<br>1 µM	0.8
Vasopressin V_{1a}	Human recombinant CHO cells	60 minutes @ RT	[^{3}H] AVP<br>0.3 nM<br>0.5 nM	AVP<br>1 µM	84.1

2. Conditions for enzyme and uptake assays conducted with Compound 3

**Target**	**Source**	**Assay conditions**	**Substrate/stimulus/tracer**	**Measured component**	**Detection method**	**Compound 3 % Inhibition @ 10 µM**
Acetylcholinesterase	Human recombinant (HEK-293 cells)	30 minutes @ RT	Acetylthiocholine (400 µM)	5 Thio 2 nitrobenzoic acid	Photometry	8.4
COX1	Human recombinant	3 minutes @ RT	Arachidonic acid (3 µM) + ADHP (25 µM)	Resorufin (oxidized ADHP)	Fluorimetry	-9.3
COX2	Human recombinant (Sf9 cells)	5 minutes @ RT	Arachidonic acid (2 µM) + ADHP (25 µM)	Resorufin (oxidized ADHP)	Fluorimetry	-18.8
IRK (InsR)	Human recombinant	10 minutes @ RT	ATP + Ulight-PolyGAT[EAY(1:1:1)]n (50 nM)	Phospho-Ulight-PolyGAT[EAY(1:1:1)]n	LANCE	-17.3
Lck kinase	Human recombinant (insect cells)	10 minutes @ RT	ATP + Ulight-PolyGAT[EAY(1:1:1)]n (25 nM)	Phospho-Ulight-PolyGAT[EAY(1:1:1)]n	LANCE	9.5
PDE3A	Human recombinant (Sf9 cells)	20 minutes @ RT	[^{3}H]cAMP + cAMP (0.5 µM)	[^{3}H]5'AMP	Scintillation counting	15.9
PDE4D2	Human recombinant (Sf9 cells)	20 minutes @ RT	[^{3}H]cAMP + cAMP (0.5 µM)	[^{3}H]5'AMP	Scintillation counting	4.4
PDE5 (non-selective)	Human platelets	60 minutes @ RT	[^{3}H]cGMP + cGMP (1 µM)	[^{3}H]cGMP	Scintillation counting	15.5

Target	Source	Assay conditions	Substrate/stimulus/tracer	Measured component	Detection method	Compound 3 % Inhibition @ 10 µM
PDGFRα kinase	Human recombinant	60 minutes @ RT	ATP + Ulight-TK peptide (100 nM)	Phospho-Ulight-TK peptide	LANCE	-2.6
PDGFRβ kinase	Human recombinant (insect cells)	30 minutes @ RT	ATP + Ulight-PolyGAT[EAY(1:1:1)]n (25 nM)	Phospho-Ulight-PolyGAT[EAY(1:1:1)]n	LANCE	-1.0
PKCα	Human recombinant (insect cells)	15 minutes @ RT	ATP + biotinyl-βAβAβ AKIQASFRGHMARKK (60 nM)	Phospho-biotinyl-βAβAβ AKIQASFRGHMARKK	HTRF	1.4
PKCε	Human recombinant (insect cells)	60 minutes @ RT	ATP + biotinyl-βAβAβ AKIQASFRGHMARKK (400 nM)	Phospho-biotinyl-βAβAβ AKIQASFRGHMARKK	HTRF	6.8

3. Conditions for functional assays conducted with Compound 3

Target	Source	Assay conditions	Reference agonist	Measured component	Detection method	% (Inhibition) of control response @ 10 µM*
Adenosine A_1 (antagonist effect)	Human recombinant (CHO cells)	28°C	CPA (1 nM)	Impedance	Cellular dielectric spectroscopy	-2.3
Adenosine A_3 (agonist effect)	Human recombinant (CHO cells)	20 minutes @ 37°C	IB-MECA (100 nM)	cAMP	HTRF	10.5
Adenosine A_3 (antagonist effect)	Human recombinant (CHO cells)	20 minutes @ 37°C	IB-MECA (10 nM)	cAMP	HTRF	8.5
Bombesin BB_1 (agonist effect)	Human recombinant (CHO cells)	RT	neuromedin B (10 nM)	Intracellular $[Ca^{2+}]$	Fluorimetry	4.4
Bombesin BB_1 (antagonist effect)	Human recombinant (CHO cells)	RT	Neuromedin B (0.1 nM)	Intracellular $[Ca^{2+}]$	Fluorimetry	28.5
C5a (agonist effect)	Human recombinant (HEK-293 cells)	20 minutes @ 37°C	hC5a (100 nM)	cAMP	HTRF	-18.1
C5a (antagonist effect)	Human recombinant (HEK-293 cells)	20 minutes @ 37°C	hC5a (10 nM)	cAMP	HTRF	-0.2
Corticotropin-releasing factor CRF_1 (agonist effect)	Human recombinant (CHO cells)	30 minutes @ 37°C	ovine CRF (1 µM)	cAMP	HTRF	-2.7

Target	Source	Assay conditions	Reference agonist	Measured component	Detection method	% (Inhibition) of control response @ 10 µM*
Corticotropin-releasing factor CRF_1 (antagonist effect)	Human recombinant (CHO cells)	30 minutes @ 37°C	Ovine CRF (10 nM)	cAMP	HTRF	55.7<br>IC_{50}: 10 µM<br>K_B: 3.6 µM
Neurokinin NK_1 (agonist effect)	U-373MG cells (endogenous)	RT	$[Sar^9,Met(O_2)^{11}]$-SP (30 nM)	Intracellular $[Ca^{2+}]$	Fluorimetry	-5.2
Neurokinin NK_1 (antagonist effect)	U373 cells (endogenous)	RT	$[Sar^9,Met(O_2)^{11}]$-SP (1 nM)	Intracellular $[Ca^{2+}]$	Fluorimetry	36.7<br>IC_{50}: 17 µM<br>K_B: 2.4 µM
Neurokinin NK_3 (agonist effect)	Human recombinant (CHO cells)	RT	$[MePhe^7]$-NKB (30 nM)	Intracellular $[Ca^{2+}]$	Fluorimetry	7.0
Neurokinin NK_3 (antagonist effect)	Human recombinant (CHO cells)	RT	$[MePhe^7]$-NKB (1 nM)	Intracellular $[Ca^{2+}]$	Fluorimetry	40.8<br>IC_{50}: 17 µM<br>K_B: 1.6 µM
TP (TXA_2/PGH_2) (agonist effect)	Human recombinant (HEK-293 cells)	RT	U 44069 (0.3 µM)	Intracellular $[Ca^{2+}]$	Fluorimetry	-2.8
TP (TXA_2/PGH_2) (antagonist effect)	Human recombinant (HEK-293 cells)	RT	U 44069 (10 nM)	Intracellular $[Ca^{2+}]$	Fluorimetry	62.3
TP (TXA_2/PGH_2) (antagonist effect)	Human recombinant (HEK-293 cells)	RT	U 44069 (10 nM)	Intracellular $[Ca^{2+}]$	Fluorimetry	55.1<br>IC_{50}: 3.6 µM<br>K_B: 0.51 µM

**Target**	**Source**	**Assay conditions**	**Reference agonist**	**Measured component**	**Detection method**	**% (Inhibition) of control response @ 10 µM***
Vasopressin V_{1a} (antagonist effect)	Human recombinant (CHO cells)	RT	AVP (10 nM)	Intracellular [Ca^{2+}]	Fluorimetry	51.9<br>IC_{50}: 9.1 µM<br>K_B: 0.31 µM
Vasopressin V_{1B} (antagonist effect)	Human recombinant (RBL cells)	RT	Vasopressin (5 nM)	Intracellular [Ca^{2+}]	Fluorimetry	7.7
Vasopressin V_2 (antagonist effect)	Human recombinant (CHO cells)	30 minutes @ RT	AVP (0.03 nM)	cAMP	HTRF	-15.1

*For agonist effect, % of reference agonist response and for antagonist effect, % inhibition of reference agonist response. If available IC_{50} and K_B values are also presented
